# Comparative gene co-expression networks show enrichment of brassinosteroid and vitamin B processes in a seagrass under simulated ocean warming and extreme climatic events

**DOI:** 10.3389/fpls.2024.1309956

**Published:** 2024-01-26

**Authors:** Mitchell W. Booth, Elizabeth A. Sinclair, Elisabeth Maria U. Jung, Rachel Austin, Philipp E. Bayer, Siegfried L. Krauss, Martin F. Breed, Gary A. Kendrick

**Affiliations:** ^1^ School of Biological Sciences, The University of Western Australia, Perth, WA, Australia; ^2^ Oceans Institute, The University of Western Australia, Perth, WA, Australia; ^3^ Kings Park Science, Department of Biodiversity, Conservation and Attractions, Perth, WA, Australia; ^4^ Minderoo Foundation, Perth, WA, Australia; ^5^ College of Science and Engineering, Flinders University, Adelaide, SA, Australia

**Keywords:** gene expression, low light stress, thermal stress, marine heatwave (MHW), RNA-Seq, *Posidonia australis*, climate change, WGCNA

## Abstract

**Introduction:**

Ocean warming combined with extreme climatic events, such as marine heatwaves and flash flooding events, threaten seagrasses globally. How seagrasses cope with these challenges is uncertain, particularly for range-edge populations of species such as *Posidonia australis* in Shark Bay, Western Australia. Analyzing gene expression while manipulating multiple stressors provides insight into the genetic response and resilience of seagrasses to climate change. We conducted a gene expression study on a polyploid clone of *P. australis* during an 18-week mesocosm experiment to assess the responses to single and combined future climate change-associated stressors.

**Methods:**

Plants were exposed to (1) future ocean warming temperature (baseline +1.5°C) followed by a simulated marine heat wave (baseline +5.5°C), (2) light deprivation simulating observed marine heatwave driven turbidity (95% shade) at baseline temperatures, or (3) both stressors simultaneously. Basal leaf meristems were sampled for gene expression analysis using RNA-seq at four time points during the experiment. Weighted gene co-expression network analysis, GO term enrichment, and KEGG pathway enrichment analyses were used to identify stress responses.

**Results:**

Shaded plants showed specific gene enrichment for shade avoidance (programmed cell death) after three weeks of stress, and before any heated tanks showed a specific heat response. Shaded plants were positively correlated with programmed cell death and stress-related processes at the end of the experiment. Once ocean warming temperatures (+1.5°C) were in effect, gene enrichment for heat stress (e.g., ROS scavenging and polyamine metabolism) was present. Vitamin B processes, RNA polymerase II processes. and light-related meristematic phase changes were expressed with the addition of simulated MHW. Heated plants showed meristematic growth signatures as well as trehalose and salicylic acid metabolism. Brassinosteroid-related processes were significantly enriched in all stressor treatments at all time points, except for the isolated heat-stressed plants three weeks after stressor initiation.

**Discussion:**

Gene expression responses to the interaction between heat waves and turbidity-induced light reduction support the observed geographical scale mortality in seagrasses observed for *P. australis* in Shark Bay, suggesting that even this giant polyploid clone will be negatively impacted by more extreme climate change projections.

## Introduction

Ecosystems are affected by global climate change (e.g., Scheffers et al., 2016; Hughes et al., 2017). Some of these changes are clearly visible and have received much public attention, such as coral bleaching events (Hughes et al., 2017), whereas other changes are more insidious and hidden. Global temperatures are projected to continue to rise throughout the 21st century with extreme climatic events becoming more common. Marine heat waves (MHWs) have been predicted to increase in frequency and intensity (Hobday et al., 2018; Oliver et al., 2018). Such extreme events can cause widespread mortality and collapse of ecosystems and their services (Wernberg et al., 2016; Pecl et al., 2017). Identifying how individuals respond to climate-induced stressors that negatively affect the resilience of species is fundamental to managing the impacts of climate change (Hoffmann and Sgrò, 2011; Lankau et al., 2011).

Environmental stressors affect plant physiology and fitness through changes in morphology, reproduction, survival, growth rates, metabolic rates, and gene expression (Hoffmann and Sgrò, 2011; Zandalinas et al., 2022). Rising ocean temperatures and reduced light availability, for example, caused by flash flooding and subsequent sediment resuspension in the water column, are two key stressors for marine plants (Duarte et al., 2018). Each stressor has an effect in isolation; however, in combination, they can have additive or synergistic effects (Brook et al., 2008; Jung et al., 2023). Interactions among stressors can push seagrass populations above their functional threshold, where range contractions and extinctions are likely to occur (York et al., 2013; Lefcheck et al., 2017; Kendrick et al., 2019). Gene expression studies have provided useful information on climate stressors in isolation, but only recently have stressors been tested in combination (Zhang and Sonnewald, 2017; Tsioli et al., 2022; Santillán-Sarmiento et al., 2023).

Genomic tools can be combined with *ex situ* mesocosm experiments to increase our understanding of the ability of a species to respond to climate change (Stillman and Armstrong, 2015). Transcriptomic studies have indicated that gene regulation plays a considerable role in phenotypic plasticity and acclimation (King et al., 2018; Rivera et al., 2021). Changes in the expression levels of candidate genes have been reported in mesocosm experiments using seagrasses, such as those associated with single stressors (Franssen et al., 2011; Franssen et al., 2014) and interactions among multiple stressors (Marín-Guirao et al., 2016; Malandrakis et al., 2017; Zhang et al., 2023). However, with the introduction of RNA-seq capabilities, measuring global transcriptomic changes provides an opportunity to gain a broader understanding of the gene networks that influence stress responses.

A polyploid *Posidonia australis* clone was recently discovered in the metahaline waters of Shark Bay (Edgeloe et al., 2022), persists across 200 km^2^, and has a wide environmental gradient (15°C–27°C, 35 PSU to 49 PSU, high light exposure, 1,500 µmol m s^−1^ to 3,000 µmol m s^−1^; Edgeloe et al., 2022). Climate change has been identified as the single largest threat to the persistence of temperate seagrasses in Shark Bay (Kendrick et al., 2019), with ~36% of meadows affected during the last extreme marine heatwave (MHW) in the Austral summer of 2010–2011 (Strydom et al., 2020).

We conducted an 18-week mesocosm experiment using the polyploid *P. australis* clone from Shark Bay to assess temporal gene expression responses to isolated and combined effects of elevated heat and light deprivation stressors in the context of an MHW under future climate change projections. We addressed the following research questions: (1) What are the effects of projected warming with climate change in combination with temperature increases associated with a MHW? (2) What are the effects of extreme reductions in light associated with projected climate change and MHW increases in storm events and flooding-induced turbidity? (3) What are the additive and/or synergistic effects of both projected increases in temperature and decreased light associated with climate-induced ocean warming and the increased frequency of marine heatwaves? We used RNA-seq and weighted gene co-expression network analysis to identify stressor-responsive genes that were significantly positively correlated with low light (shade), elevated heat (heat), and a combination of the two (heat and shade) throughout the experiment.

## Methods

### Plant collections

Ramets of the polyploid *P. australis* clone were harvested from Middle Bluff (25.823980°S, 113.464010°E) in the western gulf of Gathaagudu (in the local Malgana language), Shark Bay, northwest Western Australia, in March 2020. Approximately 350 ramets were collected via SCUBA from growing edges of the meadow at an average depth of 1.5 m. The *in situ* temperature was 26.1°C ± 0.3°C (± standard error) and salinity was 40.2 PSU ± 0.2 PSU (Practical Salinity Units) at the time of collection. The details of transportation and planting are described in Jung et al. (2023).

### Mesocosm experiment

**Figure 1:**
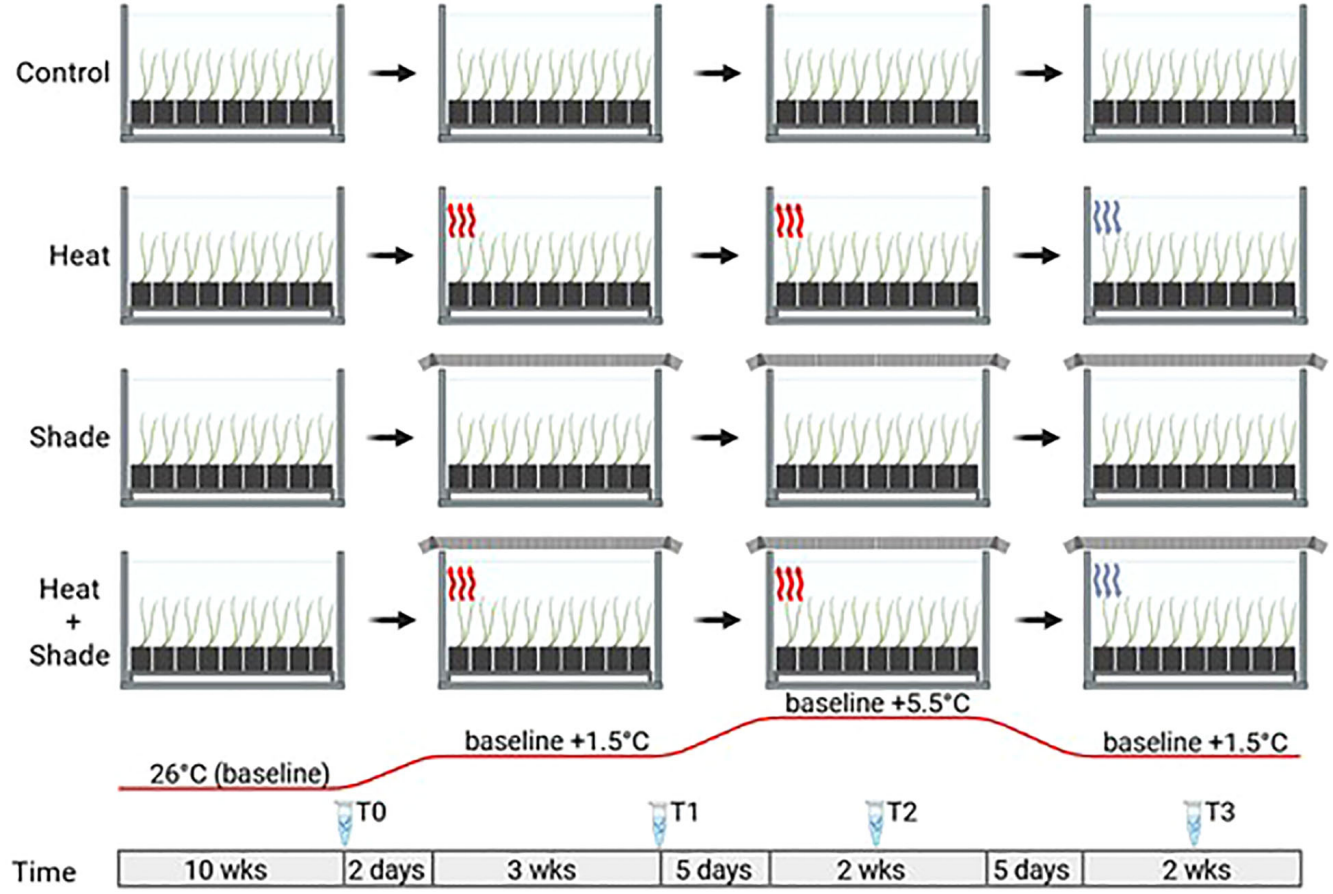
Overview of the 2010/2011 heat wave simulation under future warming mesocosm experiment. For all experimental conditions (‘Control’, ‘Heat’, ‘Shade’, and ‘Heat + Shade’) and their replicates (three replicates, n = 12 total) were allowed 10 weeks acclimation at 26°C and ambient light levels. The ‘Heat’ and ‘Heat + Shade’ tanks were then heated (red wavy lines indicate a heating event) to future warming temperature of baseline +1.5°C over two days. ‘Shade’ and ‘Heat + Shade’ tanks were also covered by shade cloth (shown by mesh covering the tanks) inducing 95% shade. Once applied, the shade cloth was not removed for the remainder of the experiment. After 3 weeks at baseline + 1.5°C, a marine heatwave was simulated by raising the ‘Heat’ and ‘Heat + Shade’ tanks to baseline +5.5°C (red wavy lines). Following 2 weeks of heatwave temperatures and five weeks of shade, the ‘Heat’ and ‘Heat + Shade’ tanks were brought back down to baseline + 1.5°C (blue wavy lines indicating a cooling event) for a recovery period of one week. Eppendorf tubes indicate RNA sampling at various time points throughout the experiment (T0: after 10 weeks of acclimation at 26°C and ambient light levels; T1: after 3 weeks of baseline + 1.5°C and/or 3 weeks of 95% shade application; T2: after 1 week of baseline + 5.5°C and/or 5 weeks of 95% shade application; T3: after 1 week of return to baseline + 1.5°C and/or 8 weeks of 95% shade application). This figure was created with BioRender.com.

A mesocosm experiment was conducted over 18 weeks (**Figure 1**). A detailed description of the mesocosm setup can be found in Jung et al. (2023). Briefly, 12 L × 1,800 L self-contained, recirculating, reinforced tanks were in a temperature-controlled glasshouse with ambient light and natural seawater. Twenty-seven pots of single *P. australis* ramets containing one apical shoot and up to four mature shoots were placed in each tank for a 10-week acclimation period to ensure sufficient time for plant adjustment to the mesocosm. The water temperature and salinity were maintained at long-term averages for the Middle Bluff of 26°C (Wijffels et al., 2018) and 42 PSU ± 3 PSU.

After the 10-week acclimation period, three tanks each were randomly assigned to either (A) ‘Control’ at a baseline temperature of 26°C and ambient light levels or one of three treatments: (B) a high temperature stressor designated ‘Heat’, (C) a low light stressor designated ‘Shade’, or (D) combined interaction between high temperature and low light stressors designated ‘Heat + Shade’ (**Figure 1**). Water temperature in the ‘Heat’ and ‘Heat + Shade’ treatments was elevated +1.5°C over two days to reach a future warming temperature simulating the projected minimum end-of-century temperature rise, equivalent to 27.5°C in Shark Bay (Geraldi et al., 2020; IPCC, 2021). The six ‘Shade’ and ‘Heat + Shade’ treatment tanks were overlaid with a double layer of high-density shade cloth to achieve 95% light deprivation simulating high turbidity levels observed in 2011 in Shark Bay, (Fraser et al., 2014). The shade cloth was not removed from ‘Shade’ or ‘Heat + Shade’ treatment tanks until the end of the experiment. Following the 3-week future warming period (baseline +1.5°C), both the ‘Heat’ and ‘Heat + Shade’ tanks were then increased by +4.0°C in +0.8°C increments over five days, simulating the +4°C increase in water temperatures experienced in Shark Bay during the 2011 heatwave (Fraser et al., 2014; Kendrick et al., 2019). After two weeks of MHW, temperatures were decreased in ‘Heat’ and ‘Heat + Shade’ tanks by −0.8°C increments over another five days, back to future warming temperatures (+1.5°C above baseline control) and held for one week to simulate a recovery period. Temperature, light, and salinity levels were monitored at the seagrass leaf canopy height throughout the experiment, as reported in the Supporting Information in Jung et al. (2023).

### Sampling for gene expression response

Three pots containing a single *P. australis* ramet with one apical shoot and up to four mature shoots were randomly harvested per tank at four time points to assess the gene expression responses. The four sampling efforts were as follows: T0: after ten weeks of acclimation at a baseline 26°C, T1: after three weeks of future warming temperature (baseline +1.5°C) and/or three weeks of shade, T2: after one week of simulated MHW (baseline +5.5°C) and/or five weeks of shade, and T3: after one week of MHW recovery under future warming temperature (baseline +1.5°C) and/or eight weeks of shade (**Figure 1**). Approximately 2 cm of basal leaf meristem tissue was collected from a single shoot, corresponding to the leaves used in Jung et al. (2023) (youngest fully developed leaf from the apical shoot), and immediately frozen in liquid nitrogen prior to storage at −80°C.

### RNA extraction, sequencing, and assembly

Approximately 100 mg of basal leaf meristem tissue was weighed for each sample (three samples from each treatment from four time points, n = 48 samples) and ground in a 2 mL screw-cap tube with six 3 mm spherical YSZ grinding media beads using a 2010 Geno/Grinder^®^ (SPEX Sample Prep, Metuchen, NJ, USA). Samples were placed in prechilled (-80°C) metal tube blocks while grinding and left for 1 min at 1,300 rpm or pulsed for 30 s until sufficiently ground into powder. The tube block was refreshed with liquid nitrogen to ensure that samples remained chilled. A Spectrum™ Plant Total RNA kit (Sigma-Aldrich^®^ St Louis, MO, USA) was used to extract total RNA from the samples, which was then quantified and treated according to the protocols described by Booth et al. (2022). RNA was sent for poly(A)-selected library preparation for mRNA sequencing via an Illumina^®^ HiSeq^®^ sequencing platform (short reads of 2 bp × 150 bp at 30× coverage) by GENEWIZ^®^ (Suzhou, China).

Raw fastQ sequence reads were initially assessed for quality using FastQC (http://www.bioinformatics.babraham.ac.uk/projects/fastqc/) and were mapped to the *P. australis* genome assembly (GCA_024699735.1; Bayer et al., 2022 preprint). Mapping was performed using HISAT2 v2.0.5 (http://daehwankimlab.github.io/hisat2/) (Kim et al., 2019) and samtools v1.9 (http://samtools.sourceforge.net/) (Li et al., 2009). The mapped reads were assembled into transcripts using the StringTie 2.1.1 (https://ccb.jhu.edu/software/stringtie/; Pertea et al., 2015). Gffcompare (https://ccb.jhu.edu/software/stringtie/gffcompare.shtml; Pertea and Pertea, 2020) was used to compare StringTie transcripts with known transcripts.

### Temporal gene co-expression networks

Weighted Gene Co-expression Network Analysis (WGCNA) (Langfelder and Horvath, 2008) was carried out using the R packages WGCNA v1.71 and DESeq2 v1.34.0 (Love et al., 2014). WGCNA creates networks of gene expression patterns based on count data generated using the publicly available Python script prepDE.py3 (http://ccb.jhu.edu/software/stringtie/dl/prepDE.py3) and custom phenotypic metadata using Microsoft Excel. Gene counts were filtered to include only genes with counts ≥15 in at least 75% of the samples, then normalized using the “vst” (variance stabilizing transformation) function of the DESeq2 package. Genes were assigned to modules based on similar expression patterns that were correlated with the experimental treatments. Four co-expression networks were created, one for each time point (T0, T1, T2, and T3) where each experimental treatment was described: 'Heat', 'Shade', 'Heat + Shade', and ‘Control’. Principal Component Analysis (PCA) was used to visually inspect the data via the R package ggplot2 v3.3.6 (Wickham and Wickham, 2016). All networks were “signed” networks where the soft-thresholding power (the power that correlations among genes are raised to reduce noise) was set to a conservative value of 18 for all networks, as recommended per WGCNA guidelines, since no soft-threshold values were appropriate (decided by simultaneously having the lowest value to meet our 0.8 threshold for scale free topology and sufficiently close to zero mean connectivity score). These settings produced conservative networks that lack a scale-free topology fit owing to interesting biological variables (groups of samples). An exception was made for the T2 network, which fit the topology and connectivity models at a value of 20 (**Supplementary Figures 1A–D**). Once the power was chosen we then used the “BlockwiseConsensusModules” function of WGCNA to detect modules and construct the networks with default values except: “TOMtype” chosen was “signed,” “maxBlockSize was 5,000 and the soft threshold given as above. All heatmaps were generated using the “CorLevelPlot” CorLevelPlot R package (Blighe, 2018) which specifies the Pearson correlation method used to calculate the correlation coefficients between module eigengenes and experimental treatments. The blue to red scale was used to designate negative to positive correlations.

### Gene ontology enrichment and KEGG pathway enrichment

Genes were extracted from modules that were shown to have a significant correlation with each given treatment in the module-treatment heatmaps for all networks. These genes were then annotated for Gene Ontology (GO) terms using PANNZER2 (http://ekhidna2.biocenter.helsinki.fi/sanspanz/; Koskinen et al., 2015) and KEGG ID via KAAS (https://www.genome.jp/tools/kaas/; Moriya et al., 2007). The R package TopGO v2.46.0 (Alexa and Rahnenfuhrer, 2021) was used to conduct GO enrichment with a node size of five for genes in their corresponding modules, to produce a list of gene ontologies (classifications of gene functional annotations) found significantly enriched in a module/treatment of interest relative to all samples. The enriched GO terms and their P-values were input to REVIGO (http://revigo.irb.hr/; Supek et al., 2011) with a SimRel cut-off value of 0.7 against the Uniprot database (https://www.uniprot.org) to generate a condensed list of enriched GO terms. The R package clusterProfiler v.4.2.2 (Wu et al., 2021) was then used to carry out pathway enrichment on the same modules of genes using Benjamini–Hochberg P-value adjustment. This process, similar to GO enrichment, produces a list of molecular pathways (classified by KEGG database pathway annotation) found to be significantly enriched in a module/treatment of interest relative to all samples.

### Comparative presence of metabolite activity

The presence of genes involved in the biological processes of metabolites of interest from Jung et al. (2023) was searched in the transcriptome generated in this study, in accordance with the sister experiment conducted by Jung et al. (2023). A custom program was made using R to search the terms: “glucose,” “trehalose,” “sucrose,” “glucopyranose,” “polyol,” “ribitol,” “dulcitol,” “erythritol,” “ribonic,” “hexaconic,” “L-pyrolglutamate,” “L-aspartate,” “l-threonine,” “serine,” “myo-inositol,” “shikimic,” “shikimate,” “isoleucine,” “alanine,” “valine,” “phenylalanine,” “putrescine,” “rhamnose,” “glycerol,” “proline,” “threonic,” “gluconic,” “aspartate,” “citric,” “fructose,” “amino acid,” “sugar,” and “methionine” to capture all relevant metabolite-related biological processes in the enriched GO terms of modules significantly positively correlated to any treatment across three exposure time points (T1–T3). The matched terms were then filtered for relevance and accuracy.

## Results

### Transcriptome sequencing and assembly

**Figure 2:**
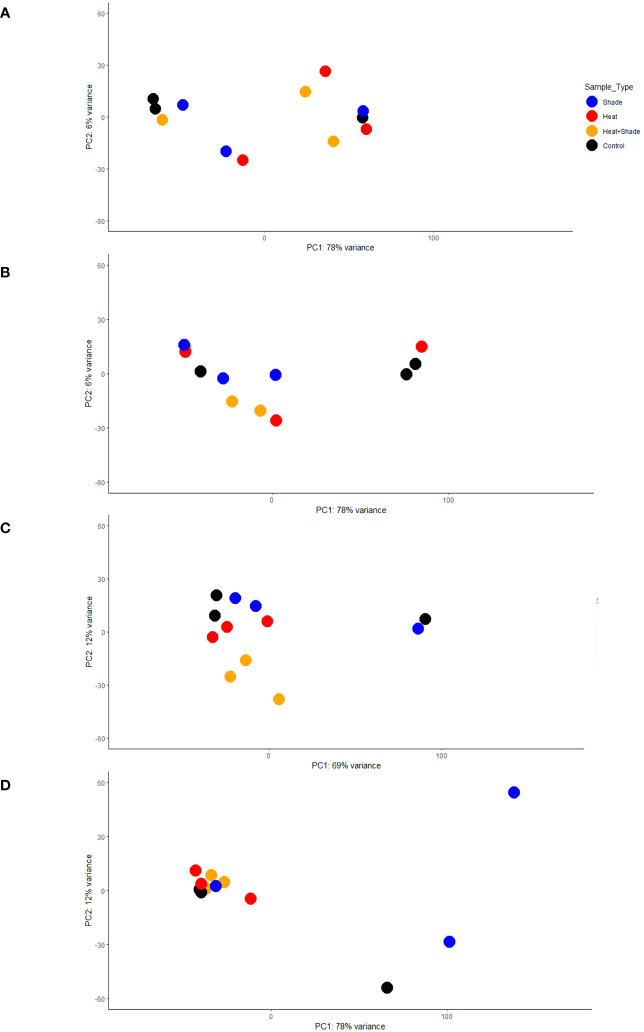
Principal component analysis (PCA) of gene expression activity over time. Each analysis dot plot shows how the variance stabilized gene expression count data of each experimental treatment relates to each other over the first two principal components. The x-axis corresponds to the largest component of variance, the first principal component (PC1); the y-axis corresponds to the second-largest component of variance, and the second principal component (PC2). The dotplots were designated by the experimental time point as follows: **(A)** T0, after 10 weeks of acclimation at 26°C and ambient light levels; **(B)** T1, after three weeks of baseline + 1.5°C and/or three weeks of 95% shade application; **(C)** T2, after one week of marine heat wave (baseline + 5.5°C) and/or five weeks of 95% shade application; and **(D)** T3, after one week of a return to baseline + 1.5°C and/or eight weeks of 95% shade application.

**Figure 3:**
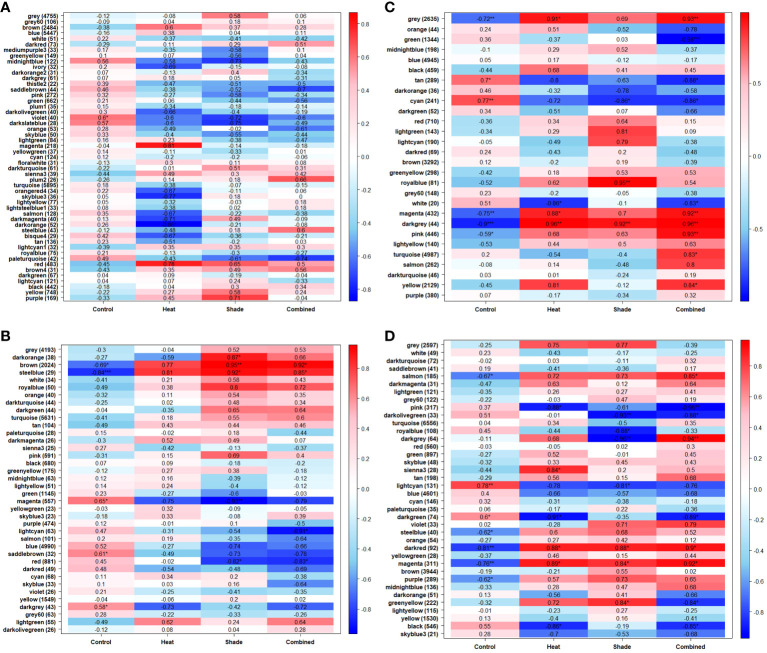
Module-treatment heatmaps show which modules of genes are most correlated with each experimental treatment. Each heatmap is a visual representation of the Weighted Gene Co-expression Network Analysis (WGCNA), showing the correlation coefficient as well as any possible significance (*P ≤0.05, **P ≤0.01, ***P ≤0.001) and either the positive (red) or negative (blue) direction of correlation. The y-axis corresponds to the modules of genes, arbitrarily named, which include several genes (shown in parentheses) grouped together by their expression pattern. The x-axis indicates the treatment type. A separate network was produced for each time point: **(A)** T0, after 10 weeks of acclimation at 26°C and ambient light levels; **(B)** T1, after three weeks of baseline + 1.5°C and/or three weeks of 95% shade application; **(C)** T2, after one week of marine heat wave (baseline + 5.5°C) and/or five weeks of 95% shade application; and **(D)** T3, after one week of a return to baseline + 1.5°C and/or eight weeks of 95% shade application.

Extracted RNA was sequenced at a depth of 20 million reads, resulting in a total of 2,069,079,918 raw 150 bp paired-end reads across all samples (**Supplementary Table 1**). FastQC analysis showed that all samples contained mean Phred scores >36, indicating that these were high-quality reads with no need for trimming and filtering owing to redundancy in downstream programs. On average, 74% of the reads for each sample were mapped to the *P. australis* genome (Bayer et al., 2022 preprint; **Supplementary Table 1**), resulting in 42,568 non-redundant genes. The assembly statistics of the *P. australis* genome used in this study contained a total size of 1,215 Mbp, an N50 score of 9,415, and 258,843 contigs longer than 1 kbp (Bayer et al., 2022 preprint). The PCA plot showed no clear clustering among the samples following a 10-week acclimation period at 26°C (T0; **Figure 2A**). There was also no significant positive correlation between any module and any treatment by WGCNA (T0, **Figure 3A**). In addition, there were no significantly enriched pathways at this time point. Approximately 28% (11,972 of 42,568) of total genes detected were significantly positively correlated to the three stressor treatments (‘Heat’, ‘Shade’, and ‘Heat + Shade’) across the sampled time points (T1–T3). Our interest was on stressor-related biological processes and the translational evidence of specific metabolites found in relation to ‘Heat’, ‘Shade’, and ‘Heat + Shade’ treatments. Therefore, only the significantly positive correlations between modules and treatments were further explored.

### Temporal change within single stressor treatments

The overall effect of the ‘Heat’ treatment was not as apparent early in the experiment (baseline +1.5°C, T1) as the ‘Shade’ treatment, as shown in comparison of T0 and T1 PCA plots (**Figures 2A, B**) where ‘Heat’ and ‘Control’ tanks remain loosely clustered in T0 (PC1 78% variance, PC2 6% variance) and T1 (PC1 78% variance, PC2 6% variance), whereas both ‘Shade’ and ‘Heat + Shade’ tanks cluster more tightly than the ‘Control’ and ‘Heat’ tanks along PC1 and PC2 of both plots. At T2 during the peak of the MHW (baseline +5.5°C), both ‘Heat’ and ‘Heat + Shade’ tanks clustered together of PC1 (69% variance) and independent of each other along PC2 (12% variance), though one replicate each of ‘Shade’ joined one replicate of ‘Control’ remaining together from T1 to T2 (**Figures 2B, C**). By the recovery period at T3, both the ‘Heat + Shade’ and ‘Heat’ replicates clustered the most tightly together along both PC1 (78% variance) and PC2 (12% variance) with two replicates of the ‘Control’ tanks and one from ‘Shade.’ A single ‘Control’ replicate remained apart from all other samples along PC1, however, now two replicates of the ‘Shade’ tanks also appear apart from all other samples along PC1, and independent of one another along PC2.

Genes modules were unique to a treatment or shared between treatments within a given time point (**Figures 3A–D**; **Supplementary Table 2**). Unique gene modules were observed for each treatment during the recovery period (T3), with some shared among all treatments (**Figure 3D**; **Supplementary Table 2**). We considered the repeated presence of significantly positively correlated with biological processes over multiple time points as a sustained effect of the stressor during the experiment. Modules found significantly positively correlated to one of the individual treatments and ‘Heat + Shade’ within the same time point were reported in the ‘Heat + Shade and Shared Modules’ section below. No modules were found significantly positively correlated in both individual treatments (‘Heat’ or ‘Shade’) and not in the ‘Heat + Shade’ treatment. Genes were considered a generalized stress response if the module was significantly positively correlated with all treatments.

### ‘Heat’ modules

No modules in the WGCNA module-treatment heatmap exhibited significantly positive correlations with ‘Heat’ after three weeks at future warming temperature (baseline +1.5°C, T1; **Figure 3B**). After three weeks of future warming plus one week of simulated MHW, three modules were observed to be significantly positively correlated with ‘Heat’ (baseline + future warming +4°C, T2): “gray”: 0.91, P <0.05; “magenta”: 0.88, P <0.05 and “darkgray”: 0.96, P < 0.01 (**Figure 3C**). These three modules were also positively correlated with the ‘Heat + Shade’ treatment and are reported in the ‘Heat + Shade and Modules Shared Within Time point’ section. After one week of MHW recovery, where temperatures were reduced to baseline +1.5°C at T3, one module was uniquely significantly positively correlated in the ‘Heat’ treatment (“sienna3”: 0.84, P <0.05; **Figure 3D**). This is the only module unique to the ‘Heat’ treatment and contains genes significantly enriched in iron homeostasis and iron transport, as well as being significantly enriched in the plant hormone signal transduction KEGG pathway (**Supplementary Table 2**).

### ‘Shade’ modules

At three weeks of exposure to 95% shade stress (T1), a single module was significantly positively correlated to the ‘Shade’ treatment (“darkorange”: 0.87, P <0.05; **Figure 3B**). The genes in this module showed enriched biological processes involved in brassinosteroid signaling and growth regulation (**Supplementary Table 2**). These terms are largely driven by a single, novel gene. KEGG pathway enrichment analysis identified the aminoacyl-tRNA biosynthesis pathway as significantly enriched within this module (**Supplementary Table 2**). At T2, after five weeks of shading, a single module was uniquely significantly positively correlated (“royalblue”: 0.95, P <0.01; **Figure 3C**). The genes in this module primarily showed enrichment for leaf senescence and related programmed cell death (**Supplementary Table 2**). By T3 (after eight weeks of shade), the single module unique to the ‘Shade’ treatment (“greenyellow”: 0.84, P <0.05; **Figure 3D**) was significantly enriched in terms involved in accumulation of chloroplasts, as well as mitochondrial respiratory chain in relation to shaded conditions.

### ‘Heat + shade’ and modules shared within time point

At T1, three weeks into the experiment at +1.5°C and 95% shading, two modules were significantly positively correlated to both the ‘Shade’ (“brown”: 0.95, P <0.01; “steelblue”: 0.92, P <0.05) and ‘Heat + Shade’ treatments (“brown”: 0.92, P <0.05; “steelblue”: 0.85, P <0.05; **Figure 3B**). No specific heat-related processes were significantly enriched in either module. The “brown” module was significantly enriched in a broad range of GO terms involved in programmed cell death, TCA cycle, and shade avoidance, along with the spliceosome KEGG pathway also found enriched in this module (**Supplementary Table 2**). Additionally, the “steelblue” module did not contain genes significantly enriched in an obvious over all biological process or pathway, with prominent terms such as “telomere maintenance,” “DNA damage response,” and “mitochondrial transmembrane transport”.

At T2 (one week of MHW and four weeks of shade exposure), three modules were significantly positively correlated to the ‘Heat + Shade’ treatment’ (“pink”: 0.93, P < 0.01; “turquoise”:0.83, P <0.05; “yellow”:0.84, P <0.05; **Figure 3C**). The “pink” module is significantly enriched in biological processes involved in RNA polymerase II transcription and translation, as well as light-related processes and meristem vegetative to reproductive phase transition with terms such as “cellular response to blue light,” “long-day photoperiodism, flowering,” “regulation of meristem growth,” “positive regulation of reproductive process,” and “vegetative to reproductive phase transition in meristem.” The “turquoise” module was found to be significantly enriched in a broad number of processes including those involved in tetrahydrofolate (vitamin B9) interconversion and purine nucleoside/nucleotide biosynthesis, as well as meristematic development and maintenance and ROS scavenging (**Supplementary Table 2**). The “yellow” module instead showed a broad range of stress related biological processes such as “regulation of cellular response to stress,” “response to endoplasmic reticulum stress,” “response to temperature synthesis” as well as calcium, gibberellic acid, and phosphate processes.

Two modules were found significantly positively correlated between both the ‘Heat’ (“gray”: 0.91, P <0.05; “magenta”: 0.88, P <0.05) and ‘Heat + Shade’ treatments (“gray”: 0.93, P <0.01; “magenta”: 0.92, P <0.01; **Figure 3C**; **Supplementary Table 2**). However, both the “gray” and “magenta” modules did not show a clear biological theme upon GO term enrichment. Lastly, the “darkgray” module was shown to be significantly positively correlated to all treatments (‘Heat’: 0.96, P <0.01; ‘Shade’: 0.92, P <0.01; and ‘Heat + Shade’: 0.96, P <0.01), and as such was characterized as a module representing a generalized stress response (**Figure 3C**). This module was found significantly enriched in the terms: “DNA topological change,” “protein glycosylation,” “brassinosteroid mediated signaling pathway,” and “response to chitin”.

At the end of a one-week recovery period at future warming temperature +1.5°C (T3) and 8 weeks of shade, two modules were significantly positively correlated to the ‘Heat + Shade’ treatment (“salmon”: 0.85, P <0.05; “darkgray”: 0.94, P <0.01; **Figure 3D**; **Supplementary Table 2**). The “salmon” module showed processes enriched in RNA transcription and translation, while other terms were involved in meristematic growth. The “darkgray” module was largely enriched in GO terms involved in the production/transport of complex sugars including carbohydrate and starch. Programmed cell death-related processes were also enriched in this module.

The “darkred” (‘Heat’: 0.88, P <0.05; ‘Shade’: 0.88, P <0.05; ‘Heat + Shade’: 0.9, P <0.05) and “magenta” (‘Heat’: 0.89, P <0.05; ‘Shade’: 0.84, P <0.05; ‘Heat + Shade’: 0.92, P <0.05) modules were both shown to be general stress response modules, being significantly positively correlated with all treatments (**Figure 3D**). The “darkred” module contained terms significantly enriched in processes involving brassinosteroid signaling, nitrogen, and lateral root morphogenesis. The “magenta” module instead was found to be significantly enriched in terms involved in general stress responses in plants, such as trehalose and salicylic acid metabolism, nitrogen, as well as RNA translation regulation.

### Comparative presence of metabolite activity

Only seven out of 103 matched terms remained after filtering for GO enrichment relevance with P <0.05, and five were found in modules that were significantly positively correlated across all treatments (**Table 1**). Sampling at T1 (+1.5°C), Glucose-6-Phosphate, the first intermediate of glucose metabolism, and S-adenosylmethionine, key in the oxidative stress response, were significantly enriched (‘Shade’, ‘Heat + Shade’). With the addition of a MHW (T2, baseline +5.5°C), Glycerol-3-Phosphate (‘Heat’, ‘Heat + Shade’), the output of the Calvin Cycle and source of carbohydrates was significantly enriched. Similar trends were observed for polyol Sorbitol (‘Heat + Shade’) which is integral for the conversion of glucose to fructose, and for L-phenylalanine (‘Heat + Shade’), a protein building block that can act as a temperature stress defense compound. After one week recovery from the MHW (baseline +1.5°C), L-phenylalanine was still significantly enriched along with S-adenosylmethionine, a precursor to ethylene production and plant senescence, and Trehalose, a protectant against abiotic stressors including heat, high salinity, and others (‘Heat’, ‘Shade’, ‘Heat + Shade’).

**Table 1:** Summary of metabolites of interest, associated gene modules, and GO enrichment terms.

Time point	Module	Treatment Significance	Term Searched/Matched Term	Matched Term and Description	Reference	GO Term Enrichment P-value
**T1**	brown	Shade (P <0.01), Heat + Shade (P <0.05)	Glucose/Glucose 6-phosphate metabolic process.	Glucose 6-phosphate is an intermediate of glucose metabolism, glycolysis, and the pentose phosphate pathway. Involved in reactive oxygen species signaling and confers plant heat stress tolerance by regulating H2O2 levels under heat stress.	Rende et al. (2019)	0.0411
**T1**	brown	Shade (P <0.01), Heat + Shade (P <0.05)	Methionine/S-adenosylmethionine metabolic process.	Methionine is a precursor of S-adenosylmethionine (SAM). It plays a key role in the initiation of mRNA translation. It is a key player in the oxidative stress response and acts as a reactive oxygen species scavenger. It controls the level of several key metabolites such as ethylene, polyamines and biotin.	Amir and Hacham (2008)	0.0227
**T2**	gray	Heat (P <0.05), Heat + Shade (P <0.01)	Glycerol/Glycerol-3-phosphate metabolic process	Glycerol-3-phosphate metabolic pathway. Is an output from the Calvin Cycle and source of carbohydrates required for cell maintenance and growth. Involved in plant defense mechanism, precursor of carbohydrate and glycerolipid biosynthesis.	Lai et al. (2015)	0.0206
**T2**	yellow	Heat + Shade (P <0.05)	Polyol/Polyol catabolic process	Sorbitol (a common polyol) is involved in a two-step metabolic pathway that converts glucose to fructose via sorbitol, creating oxidative stress.	Krasensky and Jonak (2012)	0.0126
**T2**	yellow	Heat + Shade (P <0.05)	Phenylalanine/L-phenylalanine metabolic process	L-phenylalanine is a precursor of secondary (defense) metabolism. It is critical for stress defense, UV protection, reproduction, and signaling. Phenylalanine is an essential amino acid to make proteins and other vital molecules, such as neurotransmitters and hormones.	Tohge et al. (2013); Yoo et al. (2013)	0.0252
**T3**	darkred	Heat (P <0.05), Shade (P <0.05), Heat + Shade (P <0.05)	Methionine/S-adenosylmethionine metabolic process.	Methionine is a precurser of S-adenosylmethionine (SAM). It plays a key role in the initiation of mRNA translation. It is a key player in the oxidative stress response and acts as a reactive oxygen species scavenger (ROS). It controls the level of several key metabolites such as ethylene, polyamines, and biotin.	Amir and Hacham (2008)	0.0190
**T3**	magenta	Heat (P <0.05), Shade (P <0.05), Heat + Shade (P <0.05)	Trehalose/Trehalose metabolism in response to stress	Trehalose is involved in plant growth, photosynthesis and cell wall production. Significant levels of trehalose in plants act as protectants against various abiotic stresses, including heat, drought, high salinity, and UV rays. Interacts with sucrose production.	Grennan (2007)	0.0353

The metabolite activity of interest was chosen from a study by Jung et al. (2023). The searched terms are based on the metabolite of interest and are broad enough to capture all biological activities of the metabolite when named explicitly. GO terms were searched using transcriptome data generated in this study and relevant terms that were also significantly enriched (P <0.05) in significantly correlated (P <0.05) gene modules to any experimental treatment were reported.

## Discussion

This study is one of the few that attempted to identify early warning responses that occur soon after stressor initiation via RNA-seq to climate change stressors in marine plants and is strengthened by the combination of gene expression and metabolomics in the same experiment. The largest observed changes in gene expression appeared under potential additive or synergistic stress conditions that simulated dramatic decreases in light (95% reduction) and extreme increases in temperature (baseline +5.5°C). The greater impact of synergistic rather than single stressors on biological systems has previously been reported in observations of climate change in marine (Oomen and Hutchings, 2017) and terrestrial (Zandalinas and Mittler, 2022) ecosystems. Our investigation of changes in expressed genes with projected future warming temperatures (baseline +1.5°C) was minimal, with predominantly generalized processes commonly seen in stress responses. We attribute this finding to the plasticity of natural gene expression responses to environmental stimuli, which is not indicative of a true stress response. However, genes associated with a stress response to additional warming by simulating a marine heat wave (baseline +5.5°C) showed an increased metabolic load. Post-MHW recovery was minimal, and a sustained stress response was observed across all treatments in trehalose (Sadak et al., 2019) and salicylic acid (El-Esawi et al., 2017) metabolism. These metabolites were also found in the regulation studied in the same experiment (Jung et al., 2023), and have been linked to the activation of antioxidant defense against reactive oxygen species (ROS) and the cellular stress-induced damage they cause, a common effect of almost any environmental stressor on plant health (Sewelam et al., 2016). While we acknowledge that no parameter other than RNA-seq was measured to corroborate a stress response, by the end of the experiment, we observed high mortality and/or signs of leaf damage across treatments, except in the control tanks. However, even with this conserved response, these results suggest a +5.5°C temperature increase would impact seagrass performance and may lead to increased seagrass morbidity and mortality.

The interaction between shade and heat had a unique stress response signature at 5.5°C above the baseline, involving genes for vitamin B9 biological processes. B-vitamins are metabolite precursors that break down during stress, leading to the inhibition of metabolic processes (Hanson et al., 2016). The role of vitamin B, specifically tetrahydrofolate (vitamin B9) and its one-carbon reactions, has received minimal attention in relation to plant stress, including in seagrasses. Vitamin B is important due to its role as metabolic co-factor precursors (Hanson et al., 2016). Vitamin B deficiency may be caused by abiotic stressors in plants, and tetrahydrofolate pathways have been reported to modulate gene expression under multiple stressors (e.g., salinity, drought, oxidative stress; Hanson et al., 2016). Differentially regulated vitamin B expression was also found during oxidative stress in *Arabidopsis* (Baxter et al., 2007), which is consistent with our findings. We suggest the need for further vitamin B research in seagrass stress responses, as their role may go beyond that of metabolic precursors.

A sustained response of brassinosteroid signaling-related processes was observed at all time points in almost all experimental treatments. The basal leaf meristem tissue is a hub for growth and signal transduction, and brassinosteroid signaling plays a central role in plant growth (Singh and Savaldi-Goldstein, 2015; Booth et al., 2022). However, the full context of brassinosteroid action under environmental stress is not yet fully understood (Saini et al., 2015). Brassinosteroid signaling in plants has been shown to promote plant growth, development and abiotic stress responses, in particular, they have been shown to help alleviate stress caused by low-light conditions through growth-mediated shade avoidance systems (Keuskamp et al., 2011). Similarly, brassinosteroids have been linked to higher chloroplast levels in rice, and thereby increasing heat tolerance (Pantoja-Benavides et al., 2022). Brassinosteroid metabolism was found to be upregulated in two temperate seagrasses (Nanozostera noltii and Zostera marina) which did not exhibit a strong sympatric heat response (Franssen et al., 2014). Further research is needed to understand the relative abundance and potentially protective interactions between brassinosteroids and chloroplast abundance under shading stress.

Programmed cell death and senescence responses were quite immediate, occurring in shading and the interaction between heat and shading well before any heat response. Evidence of a sustained programmed cell death/shade avoidance response in ‘Shade’ tanks is consistent with shading stress not being abated once applied. Low-light or shaded conditions can be beneficial for plant health, where short-day or shaded plants produce less ROS than under high-light conditions, which has many flow-on effects including increased resilience to thermal, drought, and biotic pathogen stressors (Roeber et al., 2021). However, in our experiment, where plants were subjected to sustained 95% shade, we suggest that programmed cell death responses are a result of circadian stress, in which detrimental ROS gradually accumulate over a prolonged period of darkness (Nitschke et al., 2016). Prolonged-dark or circadian stress studies largely involve darkness exposure, or in our case, a 95% reduction in light (shade), in the order of hours or days in established plants. In our experiment, the first time point post-stressor initiation (T1) was measured after three weeks of shading. The direct effects of prolonged darkness phenomena have been understudied, especially in monocots (Nitschke et al., 2016; Sobieszczuk-Nowicka, 2017), and should be a source of further research for other areas where turbid waters may impact seagrass meadows, such as Shark Bay (Fraser et al., 2014; Kendrick et al., 2019), or other marine environments increasingly impacted by anthropogenic disturbances and high rainfall events associated with climate change.

Transcriptomic and metabolomic results were used to verify each other to increase the reliability of enriched genes influencing the metabolome both in our study and in another recent study of the effects of heat waves on another temperate seagrass, *Z. marina* (Zhang et al., 2021). Gene enrichment under ocean warming and climate change-induced shading supported the metabolite regulation observed in the same experiment (Jung et al., 2023). Our study with *P. australis* showed that enrichment of gene expression in response to projected ocean warming and reduced light initially resulted in enrichment of genes that are associated with increased metabolic activity and oxidative stress (Glucose-6-Phosphate, S-adenosylmethionine; Amir and Hacham, 2008; Rende et al., 2019). Gene response to the addition of a marine heat wave resulted in the enrichment of genes associated with glucose metabolism and stress responses (Glycerol-3-Phosphate, Sorbitol, L-phenylalanine; Krasensky and Jonak, 2012; Yoo et al., 2013; Lai et al., 2015). No recovery from MHW was observed within one week as observed through the enrichment of stress response genes and a precursor gene for plant senescence (L-phenylalanine, trehalose, methionine; Grennan, 2007; Amir and Hacham, 2008; Yoo et al., 2013). These gene responses were consistent with the regulation of metabolites, and Jung et al. (2023) reported an increase in Glycerol-3-Phosphate, L-phenylalanine and a decrease in trehalose as a result of shade alone or in combination with MHW stress. Similarly, observations of seagrass shoot thinning in *P. australis* and defoliation in another temperate seagrass (*Amphibolis antarctica*) (Fraser et al., 2014; Kendrick et al., 2019) from the additive effects of baseline +5.5°C warming and turbidity-driven light deprivation generated in the 2010–2011 extreme MHW in Shark Bay are consistent with transcriptomic and metabolomic results from this mesocosm experiment. In combination, these stressors can result in geographical scale mortality in seagrasses (Fraser et al., 2014), where the greatest losses occur when extreme heating persists for ≥94 days (Strydom et al., 2020).

We examined gene expression in a single polyploid *P. australis* clone from the northern (warm) range edge of Western Australia. Individual shoots from this clone had a low range of phenotypic variation, with 28% of the transcribed genes significantly correlated to modules that correlated with the experimental stressors. It has been suggested that the clone originated as a result of historical autopolyploidy, with more recent gene loss through rediploidization, although high heterozygosity levels (~90%) are more consistent with hybridization with other *Posidonia* species through allopolyploidy (Edgeloe et al., 2022). In any case, our results suggest that the genome of this *P. australis* clone may lack vital genes or regulation among the additional gene copies needed to survive prolonged shade or combined heat and shade. This clone was showing early signs of recovery in ‘Heat’ treatment tanks by T3; however, a longer recovery time would be required to gain a better understanding of recovery capacity. Whether this example of polyploidy in a seagrass is more tolerant to abiotic stressors representative of *P. australis* more widely (Sinclair et al., 2023) and/or its diploid progenitors (see Edgeloe et al., 2022), as polyploidy is often thought to confer (Van de Peer et al., 2021; Heslop-Harrison et al., 2023), requires further comparative study in this context with the neighboring diploid *P. australis*.

In conclusion, brassinosteroids were consistently enriched throughout the heat and/or shade stress responses. We observed that this *P. australis* clone in Shark Bay could withstand future ocean warming temperatures (+1.5°C) but showed a significant transcriptomic response to the additional pressure of an extreme climatic event (MHW). The effect of prolonged shading was immediate and extreme, with stressor-induced damage control being a major theme in the correlated gene expression. This damage response was further exacerbated by the interaction of ‘Heat’ and ‘Shade’, inducing a novel vitamin B gene expression response. However, meaningful plant recovery was not observed at the end of this experiment. Therefore, it is unlikely that this *P. australis* clone would survive extreme marine heatwave conditions (Hobday et al., 2018) under future ocean warming. Further research into the role of brassinosteroids and vitamin B in relation to plant stress responses will be important for understanding the resilience of *P. australis* over longer recovery phases.

## Data availability statement

The datasets presented in this study can be found in online repositories. The names of the repository/repositories and accession number(s) can be found below: https://www.ncbi.nlm.nih.gov/, PRJNA1025046.

## Author contributions

MWB: Conceptualization, Data curation, Formal analysis, Funding acquisition, Investigation, Methodology, Project administration, Software, Validation, Visualization, Writing – original draft, Writing – review & editing. EAS: Conceptualization, Funding acquisition, Investigation, Methodology, Project administration, Resources, Supervision, Writing – original draft, Writing – review & editing. EMUJ: Conceptualization, Methodology, Project administration, Writing – review & editing. RA: Methodology, Project administration, Writing – review & editing. PEB: Data curation, Funding acquisition, Resources, Software, Writing – review & editing. SLK: Conceptualization, Funding acquisition, Methodology, Writing – review & editing. MFB: Conceptualization, Funding acquisition, Investigation, Methodology, Project administration, Supervision, Writing – review & editing. GAK: Conceptualization, Funding acquisition, Investigation, Methodology, Project administration, Supervision, Writing – review & editing.
