# Supplementary material for: Comparative gene co-expression networks show enrichment of brassinosteroid and vitamin B processes in a seagrass under simulated ocean warming and extreme climatic events
Source: Front Plant Sci. 2024 Jan 26;15:1309956. doi: 10.3389/fpls.2024.1309956 (PMC10853371; doi:10.3389/fpls.2024.1309956)
Supplement: Supplementary file 1 [file DataSheet_1.pdf]

A)

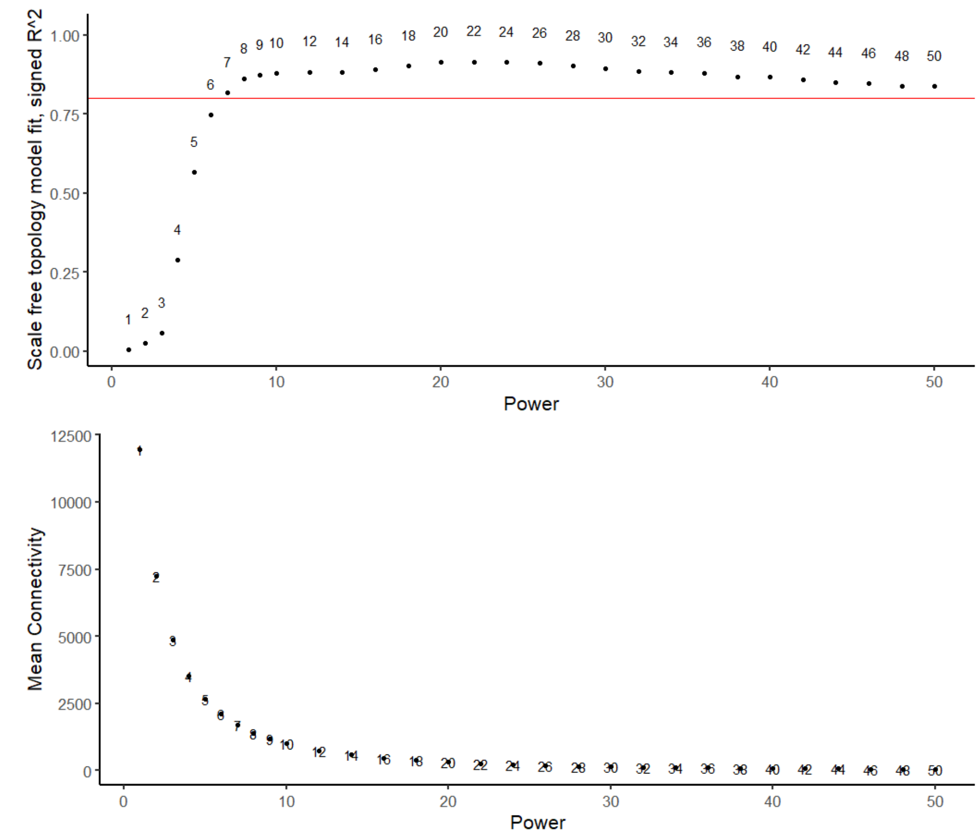
Scale free topology model fit, signed R^2
1.00
0.75
0.50
0.25
0.00
Power
Mean Connectivity
12500
10000
7500
5000
2500
0
Power

B)

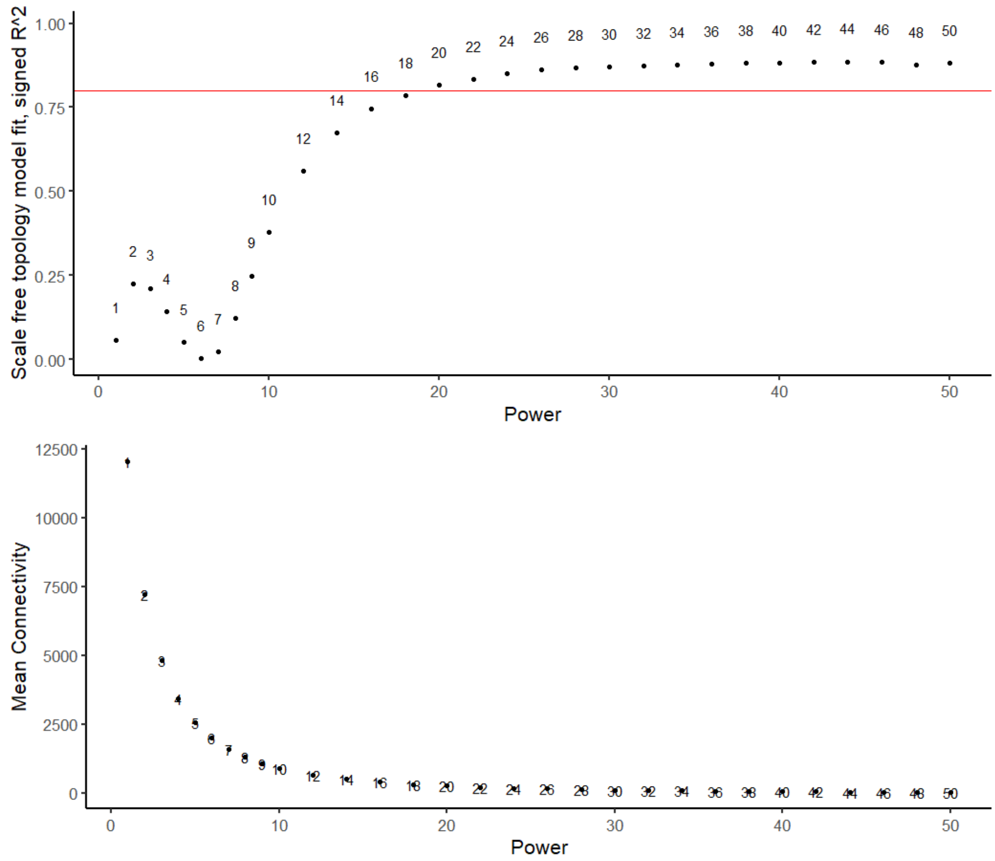
Scale free topology model fit, signed R^2
1.00
0.75
0.50
0.25
0.00
Power
Mean Connectivity
12500
10000
7500
5000
2500
0
Power

C)

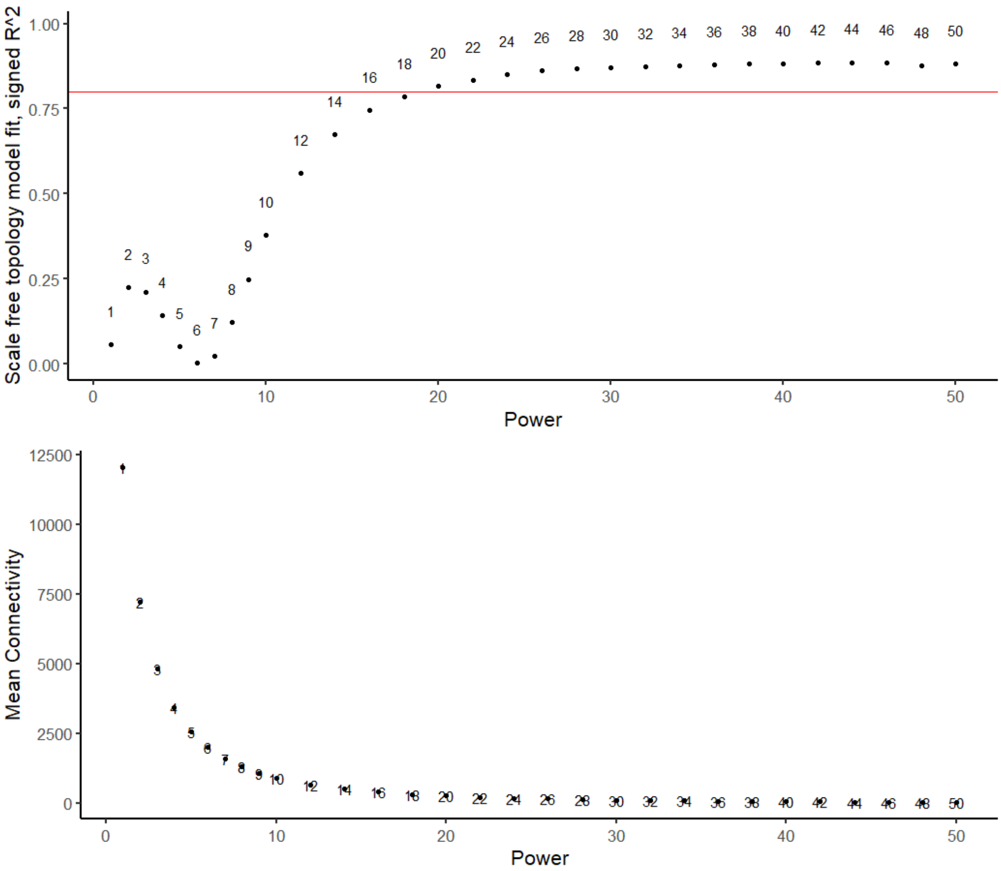
Scale free topology model fit, signed R^2
1.00
0.75
0.50
0.25
0.00
Power
Mean Connectivity
12500
10000
7500
5000
2500
0
0
10
20
30
40
50
Power

D)

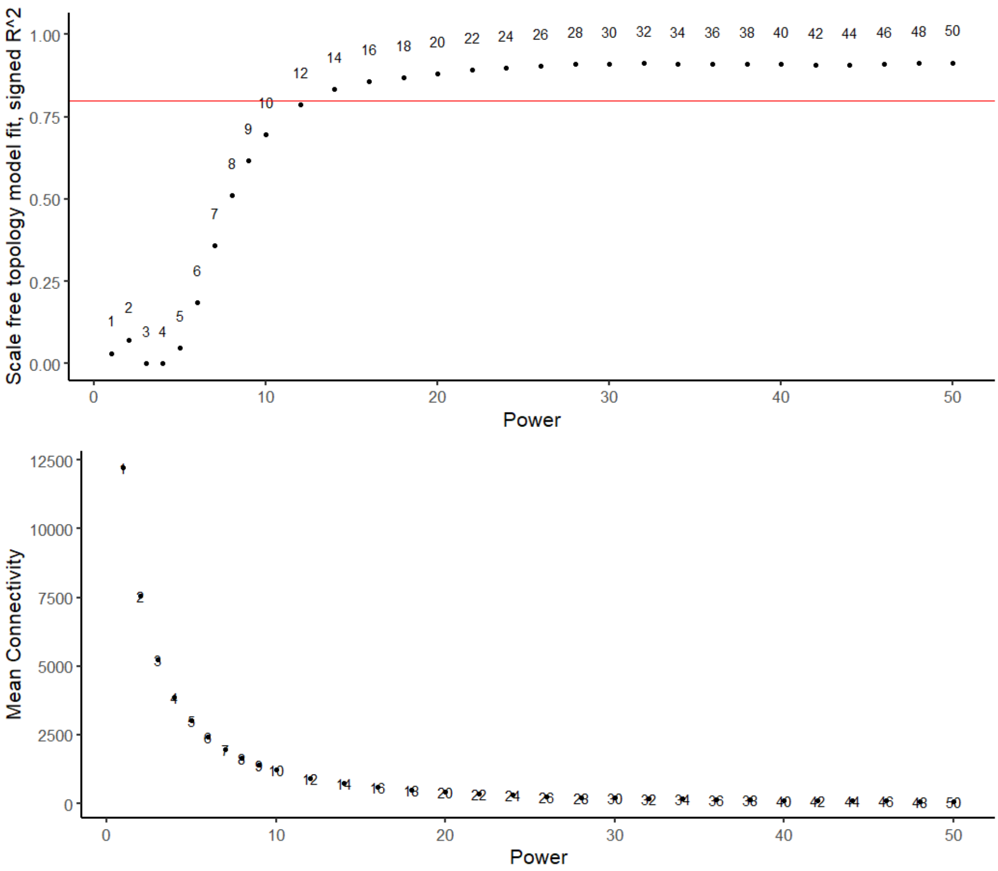
Scale free topology model fit, signed R^2
1.00
0.75
0.50
0.25
0.00
Power
Mean Connectivity
12500
10000
7500
5000
2500
0
0
10
20
30
40
50
Power

Supplementary Figure S1. Scale free topology and mean connectivity plots used to decide the soft-thresholding power. The scale free topology R^2 values and mean connectivity values are shown at each designation of power (0 - 50). The signed networks soft-thresholding power is chosen by having a signed R^2 value above the 0.8 threshold for scale free topology (red line) and a simultaneously low mean connectivity score. The resulting soft thresholding power was chosen for constructing modules. **(A)** T0, after 10 weeks acclimation at 26 °C and ambient light levels, **(B)** T1, after 3 weeks of baseline + 1.5 °C and/or 3 weeks + 2 days of 95% shade application, **(C)** T2, after 1 week of baseline + 5.5 °C and/or 5 weeks of 95% shade application, and **(D)** T3, after 1 week of return to baseline + 1.5 °C and/or 7 weeks + 5 days of 95 % shade. Note the soft-threshold power was set to 18 for A, B, and D and 20 for C.
