# Supplementary material for: Comparative gene co-expression networks show enrichment of brassinosteroid and vitamin B processes in a seagrass under simulated ocean warming and extreme climatic events
Source: Front Plant Sci. 2024 Jan 26;15:1309956. doi: 10.3389/fpls.2024.1309956 (PMC10853371; doi:10.3389/fpls.2024.1309956)
Supplement: Supplementary file 2 [file DataSheet_2.pdf]

Supplementary Table S1. Sample metadata from all raw read files. Read files can be identified by unique Sample IDs for each Time point (T0 - T3) and Treatment type (Control, Heat, Shade, Heat + Shade). Metadata used for quality control were generated using FastQC, with alignment to the publicly available *P. australis* genome (Bayer *et al.*, 2022 preprint).

Sample ID	Time point	Treatment type	Number of shared reads	Read length (bp)	Total bp (Gbp)	Estimated coverage (1.1Gbp)	Average base pair quality	Percent aligned with *P. australis* genome
S0C6-18-LEG7765_L1	0	Control	40578446	150	6.09	5.53	36.15	77.89
S0C11-11-LEG7763_L1	0	Control	45284666	150	6.79	6.18	36.14	75.70
S0C2-2-LEG7764_L1	0	Control	40709862	150	6.11	5.55	36.13	77.46
S0S10-16-LEG7768_L1	0	Shade	41019862	150	6.15	5.59	36.08	77.23
S0S1-2-LEG7766_L1	0	Shade	40682130	150	6.10	5.55	36.13	77.30
S0S4-9-LEG7767_L1	0	Shade	42510274	150	6.38	5.80	36.07	76.65
S0H7-6-LEG7770_L1	0	Heat	43182828	150	6.48	5.89	36.06	79.01
S0H3-25-LEG7771_L1	0	Heat	41023426	150	6.15	5.59	36.10	70.43
S0H9-5-LEG7769_L1	0	Heat	42577350	150	6.39	5.81	35.99	74.39
S0B5-4-LEG7772_L1	0	Heat + Shade	43107600	150	6.47	5.88	36.06	70.41
S0B8-6-LEG7774_L1	0	Heat + Shade	43826758	150	6.57	5.98	36.06	72.68
S0B12-15-LEG7773_L1	0	Heat + Shade	43268878	150	6.49	5.90	36.10	71.08
2C11-7-LEG7777_L1	1	Control	43077010	150	6.46	5.87	35.88	69.14
2C6-7-LEG7776_L1	1	Control	42400438	150	6.36	5.78	36.02	73.06
2C2-21-LEG7775_L1	1	Control	42177402	150	6.33	5.75	35.92	67.39
2S1-14-LEG7778_L1	1	Shade	42609236	150	6.39	5.81	35.98	72.17
2S10-4-LEG7780_L1	1	Shade	43621634	150	6.54	5.95	36.14	75.29
2S4-6-LEG7779_L1	1	Shade	44784544	150	6.72	6.11	36.16	77.75
2H9-27-LEG7781_L1	1	Heat	42072670	150	6.31	5.74	36.12	69.78
2H7-21-LEG7782_L1	1	Heat	40567268	150	6.09	5.53	36.12	77.36
2H3-23-LEG7783_L1	1	Heat	40246216	150	6.04	5.49	36.06	75.53
2B8-4-LEG7786_L1	1	Heat + Shade	44066456	150	6.61	6.01	35.94	73.41
2B5-22-LEG7784_L1	1	Heat + Shade	41566960	150	6.24	5.67	36.05	73.59
2B12-3-LEG7816_L1	1	Heat + Shade	43773380	150	6.57	5.97	36.13	73.65
3C6-24-LEG7788_L1	2	Control	43520952	150	6.53	5.93	36.13	76.47
3C11-18-LEG7789_L1	2	Control	42703458	150	6.41	5.82	36.11	75.42
3C2-27-LEG7787_L1	2	Control	40603200	150	6.09	5.54	36.05	66.76
3S4-21-LEG7791_L1	2	Shade	43642444	150	6.55	5.95	36.09	73.78
3S1-20-LEG7790_L1	2	Shade	43281882	150	6.49	5.90	36.14	73.12
3S10-18-LEG7792_L1	2	Shade	42001652	150	6.30	5.73	36.02	71.59
3H9-8-LEG7793_L1	2	Heat	42873212	150	6.43	5.85	36.01	69.95
3H3-13-LEG7795_L1	2	Heat	43727882	150	6.56	5.96	35.99	69.67
3H7-7-LEG7794_L1	2	Heat	43350358	150	6.50	5.91	35.96	75.43
3B5-2-LEG7800_L1	2	Heat + Shade	43694726	150	6.55	5.96	36.14	80.39
3B5-9-LEG7796_L1	2	Heat + Shade	46369084	150	6.96	6.32	36.15	79.22
3B12-18-LEG7798_L1	2	Heat + Shade	41958572	150	6.29	5.72	36.12	76.37
5C11-25-LEG7804_L1	3	Control	44572874	150	6.69	6.08	36.10	78.41
5C2-1-LEG7802_L1	3	Control	42824212	150	6.42	5.84	36.11	77.34
5C6-5-LEG7803_L1	3	Control	42588522	150	6.39	5.81	36.05	77.04
5S4-10-LEG7806_L1	3	Shade	43256432	150	6.49	5.90	36.07	79.80
5S10-1-LEG7807-RL1_L2	3	Shade	43190834	150	6.48	5.89	36.14	67.82
5S1-11-LEG7805-RL1_L1	3	Shade	41484056	150	6.22	5.66	36.27	68.96
5H7-23-LEG7809_L1	3	Heat	44206636	150	6.63	6.03	36.09	75.52
5H9-7-LEG7808_L1	3	Heat	46697380	150	7.00	6.37	36.11	74.63
5H3-24-LEG7810_L1	3	Heat	43977888	150	6.60	6.00	36.04	72.72
5B12-19-LEG7812_L1	3	Heat + Shade	42926388	150	6.44	5.85	35.97	72.85
5B12-27-LEG7815_L1	3	Heat + Shade	55654294	150	8.35	7.59	36.13	76.00
5B12-24-LEG7814_L1	3	Heat + Shade	41237686	150	6.19	5.62	36.09	74.14
