# Supplementary material for: Comparative gene co-expression networks show enrichment of brassinosteroid and vitamin B processes in a seagrass under simulated ocean warming and extreme climatic events
Source: Front Plant Sci. 2024 Jan 26;15:1309956. doi: 10.3389/fpls.2024.1309956 (PMC10853371; doi:10.3389/fpls.2024.1309956)
Supplement: Supplementary file 3 [file DataSheet_3.pdf]

Supplementary Table S2. Enriched GO terms and their functions for significantly correlated modules at three sampling timepoints (T1 - T3). The Enrichment values generated from TopGO and the online tool REVIGO are shown for each GO term. Each module is arbitrarily given a colour identifier for delineation of differently expressed patterns of gene expression among experimental treatments. Significantly enriched GO terms were produced from genes with membership to that module. The Sampling Timepoint and Treatment headers show which time point the coloured module is referring to. The GO ID and associated biological process are listed by sampling time point (T1 – T3) with the following parameters from TopGO: Annotated = Number of unique transcripts annotated across meristems; Significant = Number of significantly differentially expressed transcripts annotated; Expected = Number of transcripts expected to be annotated with a node size of 5; P-value = P-value calculated from the gene enrichment analysis and REVIGO: Log10(P-value); LogSize = Log10 Number of annotations in selected species (whole Uniprot database by default) in the EBI GOA database); Frequency = Proportion of selected GO term in the EBI GOA database for the selected species (whole Uniprot database by default), a higher value implies more general terms and a lower value implies more specific terms; Uniqueness = Measures whether the term is an outlier when compared semantically to the whole list of GO terms provided. Calculated as 1-(average semantic similarity of a term to all other terms), more unique terms tend to be less dispensable; Dispensability = Semantic similarity threshold (calculated using SimRel at c=0.7) at which the term was removed from the list and assigned to a cluster. Cluster representatives always have dispensability less than the user-specified 'allowed similarity' cutoff; Representative GO ID = Cluster representative (null denotes the GO term is its own cluster representative) at c = 0.7 (medium).

				TopGO				REVIGO					
**Sampling timepoint**	**Treatment**	**GO term ID**	**Biological processes**	**Annotated**	**Significant**	**Expected**	**P-value**	**Log10 (P-value)**	**LogSize**	**Frequency**	**Uniqueness**	**Dispensability**	**Representative GO ID**
**T1**	**Shade**	**"darkorange" module**											
		GO:000974	response to brassinosteroid	27	1	0.02	0.0192	-1.716698771	3.758457689	0.019068994	1	0	null
		GO:000982	unidimensional cell growth	29	1	0.02	0.0206	-1.68613278	3.502700175	0.010580581	0.991129724	0.0064674	null
		GO:001810	peptidyl-tyrosine phosphorylation	33	1	0.02	0.0234	-1.630784143	3.657533888	0.015114165	0.704638954	0.36811835	null
		GO:003048	tRNA methylation	9	1	0.01	0.0064	-2.193820026	4.889368943	0.257815593	0.735448408	0	null
		GO:004677	protein autophosphorylation	18	1	0.01	0.0128	-1.89279003	4.217931169	0.054935201	0.693696823	0.29447586	null
**T1**	**Shade, Combined**	**"brown" module**											
		GO:000039	mRNA splicing, via spliceosome	57	7	2.35	0.0188	-1.725842151	5.182759782	0.506643846	0.821966635	0.53789193	null
		GO:000096	mitochondrial RNA processing	15	3	0.62	0.0218	-1.661543506	3.35679046	0.007560409	0.867021726	0.39326839	null
		GO:000218	cytoplasmic translation	12	3	0.49	0.0115	-1.93930216	4.558924464	0.120464287	0.831781718	0.3272569	null
		GO:000609	glycolytic process	18	3	0.74	0.0356	-1.448550002	5.209225388	0.538478722	0.831756715	0.49821893	null
		GO:000663	fatty acid metabolic process	75	7	3.09	0.0403	-1.394694954	5.50943507	1.074928474	0.848812692	0.72297619	GO:0006096
		GO:000609	tricarboxylic acid cycle	12	3	0.49	0.0115	-1.93930216	5.211155262	0.540876898	0.938103873	0.06330878	null
		GO:000630	DNA methylation	7	2	0.29	0.0309	-1.510041521	4.734415753	0.18044863	0.828765052	0.63004819	null
		GO:000631	DNA recombination	39	5	1.61	0.0446	-1.350665141	5.697388904	1.657043361	0.841477354	0.2616187	null
		GO:000654	glutamine metabolic process	8	2	0.33	0.0402	-1.395773947	5.263851387	0.610653517	0.851824878	0.1569581	null
		GO:000659	polyamine biosynthetic process	6	2	0.25	0.0227	-1.643974143	4.551706107	0.118478557	0.87859936	0.13617068	null
		GO:000662	protein targeting to mitochondrion	7	3	0.29	0.0021	-2.677780705	4.44345075	0.092338103	0.830249458	0.00744126	null
		GO:004474	protein transmembrane import into intracellular organelle	5	2	0.21	0.0156	-1.806875402	4.486657131	0.101997332	0.851530045	0.76168114	GO:0006626
		GO:006500	intracellular protein transmembrane transport	13	3	0.54	0.0399	-1.399027104	4.884455496	0.254915164	0.849340776	0.81972554	GO:0006626
		GO:000678	protoporphyrinogen IX biosynthetic process	7	2	0.29	0.0309	-1.510041521	4.789446737	0.204826208	0.866353007	0.25375216	null
		GO:000687	intracellular calcium ion homeostasis	8	2	0.33	0.0402	-1.395773947	4.336819829	0.072234667	1	0	null
		GO:000705	chromosome segregation	19	2	0.78	0.0412	-1.385102784	5.079159531	0.399118402	0.989757241	0.00837442	null
		GO:000821	protein alkylation	24	4	0.99	0.0497	-1.303643611	4.901142158	0.264900358	0.866967112	0.38616464	null
		GO:000905	macromolecule biosynthetic process	1356	45	55.86	0.0456	-1.341035157	6.661562823	15.25838504	0.873522204	0.22109794	null
		GO:000906	amino acid catabolic process	17	3	0.7	0.0306	-1.514278574	5.328267447	0.708290226	0.82627979	0.67726374	null
		GO:000911	nucleotide metabolic process	81	6	3.34	0.0409	-1.388276692	6.039865234	3.64594975	0.8020759	0.38071782	null
		GO:000964	photoperiodism	23	4	0.95	0.0408	-1.389339837	3.308991029	0.006772104	0.991464369	0.2010172	null
		GO:000984	seed germination	26	4	1.07	0.0206	-1.68613278	3.057285644	0.003791846	0.975010276	0	null
		GO:000991	positive regulation of flower development	10	3	0.41	0.0067	-2.173925197	2.587710965	0.001283906	0.924119092	0.13886387	null
		GO:001002	wax biosynthetic process	6	2	0.25	0.0227	-1.643974143	2.503790683	0.001057725	0.940993801	0.0320939	null
		GO:001038	cell wall polysaccharide metabolic process	41	3	1.69	0.0411	-1.386158178	4.576156995	0.125340468	0.910392613	0.09210539	null
		GO:001049	proteasomal protein catabolic process	58	6	2.39	0.0224	-1.649751982	5.0271987	0.354111851	0.85227818	0.11842186	null
		GO:001657	histone modification	43	6	1.77	0.0153	-1.815308569	5.052155067	0.375056811	0.863331567	0.39969794	null
		GO:002261	protein-RNA complex assembly	30	5	1.24	0.007	-2.15490196	5.020663692	0.348823224	0.949661845	0.3723731	null
		GO:003112	RNA 3'-end processing	15	4	0.62	0.0012	-2.920818754	4.80756885	0.213554107	0.833356449	0	null
		GO:003298	protein-containing complex disassembly	7	2	0.29	0.0309	-1.510041521	4.853971841	0.237635655	0.950420538	0.60337222	null
		GO:003447	ncRNA processing	81	7	3.34	0.0224	-1.649751982	5.906238042	2.680299617	0.796265148	0.69071098	null
		GO:003497	response to endoplasmic reticulum stress	20	3	0.82	0.0226	-1.645891561	4.571825249	0.124096476	0.982196526	0.00761256	null
		GO:004306	regulation of programmed cell death	8	2	0.33	0.0402	-1.395773947	5.012567288	0.342380412	0.92595551	0.21514464	null

		GO:004341	macromolecule methylation	51	8	2.1	0.0032	-2.494850022	5.594848707	1.308559407	0.875967168	0.11271017	null
		GO:004593	positive regulation of nucleobase-containing compound metabolic process	72	2	2.97	0.0415	-1.381951903	5.411979785	0.858863101	0.866970282	0.47437271	null
		GO:001060	positive regulation of macromolecule metabolic process	89	3	3.67	0.0415	-1.381951903	5.610810224	1.357547397	0.866651021	0.92692856	GO:0045935
		GO:004650	S-adenosylmethionine metabolic process	6	2	0.25	0.0227	-1.643974143	4.439000924	0.091396794	0.947350926	0.05474683	null
		GO:004873	system development	284	22	11.7	0.0394	-1.404503778	5.508815115	1.073395105	0.975010276	0.59097394	null
		GO:005079	regulation of catalytic activity	117	11	4.82	0.0021	-2.677780705	5.306945868	0.67435653	0.926642733	0	null
		GO:005105	regulation of DNA metabolic process	12	3	0.49	0.0115	-1.93930216	4.836330446	0.228175997	0.911581258	0.20377506	null
		GO:005115	glucose 6-phosphate metabolic process	9	2	0.37	0.0411	-1.386158178	4.843369837	0.231904646	0.923138431	0.56608329	null
		GO:006025	regulation of macromolecule metabolic process	754	35	31.06	0.0222	-1.653647026	6.58442011	12.77514499	0.88099918	0.52013055	null
		GO:007142	malate transmembrane transport	6	2	0.25	0.0227	-1.643974143	3.485721426	0.010174787	0.907584029	0.32536835	null
		GO:000333	amino acid transmembrane transport	8	2	0.33	0.0402	-1.395773947	4.996191963	0.329710989	0.884660008	0.71179347	GO:0071423
		GO:008013	regulation of cellular response to stress	5	2	0.21	0.0156	-1.806875402	4.685992393	0.161409572	0.909965281	0.19760247	null
		GO:000996	negative regulation of signal transduction	26	4	1.07	0.0216	-1.665546249	4.993259831	0.327492427	0.905940199	0.7063063	GO:0080135
		GO:009015	establishment of protein localization to membrane	17	3	0.7	0.0226	-1.645891561	4.990849228	0.325679658	0.866345026	0.54112035	null
		GO:007265	protein localization to membrane	24	5	0.99	0.0304	-1.517126416	5.058763762	0.380807778	0.866420643	0.96559918	GO:0090150
**T1**	**Shade,**	**"steelblue" module**											
	**Combined**	GO:000072	telomere maintenance	11	1	0.01	0.0059	-2.229147988	4.5707647	0.123793794	0.839570029	0.00719703	null
		GO:000697	DNA damage response	81	1	0.04	0.0429	-1.367542708	5.894540353	2.609069457	0.984377808	0.00945334	null
		GO:004474	protein transmembrane import into intracellular organelle	5	1	0	0.0027	-2.568636236	4.486657131	0.101997332	0.480178169	0	null
		GO:000662	protein targeting to mitochondrion	7	1	0	0.0038	-2.420216403	4.44345075	0.092338103	0.423033447	0.76168114	GO:0044743
		GO:006500	intracellular protein transmembrane transport	13	1	0.01	0.007	-2.15490196	4.884455496	0.254915164	0.499129463	0.81972554	GO:0044743
		GO:004589	positive regulation of DNA-templated transcription	61	1	0.03	0.0324	-1.48945499	5.337678849	0.723806859	1	0	null
		GO:199054	mitochondrial transmembrane transport	8	1	0	0.0043	-2.366531544	4.32467335	0.070242284	0.77576531	0.2444234	null
**T2**	**Heat,**	**"darkgrey" module**											
	**Shade,**	GO:000626	DNA topological change	6	1	0	0.0043	-2.366531544	4.838395677	0.229263658	0.926309292	0	null
	**Combined**	GO:000648	protein glycosylation	55	1	0.04	0.0388	-1.411168274	5.276259546	0.628352125	0.930574707	0.10601099	null
		GO:000974	brassinosteroid mediated signaling pathway	13	1	0.01	0.0093	-2.031517051	3.724357804	0.017628758	0.685970569	0.50865968	null
		GO:001020	response to chitin	7	1	0.01	0.005	-2.301029996	2.481442629	0.001004507	0.689641844	0	null
**T2**	**Heat,**	**"grey" module**											
	**Combined**	GO:000218	cytoplasmic translation	12	3	0.4	0.00631	-2.199970641	4.558924464	0.120464287	0.88823881	0.33211726	null
		GO:000607	glycerol-3-phosphate metabolic process	7	2	0.23	0.02055	-1.687188174	4.55874456	0.120414394	0.900897561	0.54304128	null
		GO:000659	polyamine biosynthetic process	6	2	0.2	0.015	-1.823908741	4.551706107	0.118478557	0.915840279	0.25731439	null
		GO:000674	glutathione metabolic process	9	2	0.3	0.03373	-1.471983659	4.758919458	0.190922773	0.975815801	0.04663874	null
		GO:000683	dicarboxylic acid transport	11	2	0.36	0.04935	-1.306712843	4.258541507	0.060320287	0.92021559	0.63910522	null
		GO:000687	intracellular calcium ion homeostasis	8	2	0.27	0.02681	-1.571703186	4.336819829	0.072234667	1	0	null
		GO:000922	thiamine biosynthetic process	5	2	0.17	0.01022	-1.990549104	4.867178648	0.24497321	0.868837761	0.210181	null
		GO:000929	mRNA transcription	7	2	0.23	0.02055	-1.687188174	3.403292145	0.008415237	0.894137327	0.50512966	null
		GO:000961	response to bacterium	50	4	1.66	0.01461	-1.835349784	4.642622776	0.146069226	0.903875731	0.21623735	null
		GO:000964	photoperiodism	23	3	0.76	0.03291	-1.482672118	3.308991029	0.006772104	0.964209483	0.17978016	null
		GO:000986	pollen tube growth	11	2	0.36	0.04935	-1.306712843	3.149527014	0.004689915	0.972715822	0.37335543	null
		GO:001005	xylem and phloem pattern formation	10	2	0.33	0.04126	-1.384470776	2.94792362	0.002946996	0.976801413	0	null
		GO:001569	inorganic anion transport	63	3	2.09	0.0497	-1.303643611	5.447579713	0.932231995	0.953555495	0.29526131	null
		GO:001571	organic anion transport	40	5	1.33	0.03402	-1.468265691	5.450792406	0.939153777	0.928373453	0	null
		GO:001580	L-amino acid transport	8	2	0.27	0.02681	-1.571703186	4.264747008	0.06118842	0.92016846	0.43628525	null
		GO:001631	dephosphorylation	37	4	1.23	0.00361	-2.442492798	5.30807756	0.67611608	0.910132185	0.43725896	null
		GO:002261	protein-RNA complex assembly	30	4	0.99	0.03434	-1.464199709	5.020663692	0.348823224	0.994494334	0.01121981	null
		GO:003024	cellulose catabolic process	10	2	0.33	0.04126	-1.384470776	4.520693096	0.110312783	0.963702395	0.09254276	null
		GO:003048	tRNA methylation	9	3	0.3	0.00259	-2.586700236	4.889368943	0.257815593	0.894969822	0.07984036	null
		GO:004000	growth	80	5	2.65	0.03289	-1.482936127	4.457927291	0.095468039	1	0	null
		GO:004254	response to hydrogen peroxide	12	3	0.4	0.00631	-2.199970641	3.736077637	0.018111054	0.953142961	0	null
		GO:004259	response to starvation	18	2	0.6	0.03306	-1.480697151	4.354876423	0.075301405	0.931151403	0.56349405	null
		GO:004364	inositol phosphate metabolic process	8	2	0.27	0.02681	-1.571703186	4.044186851	0.036820821	0.870697238	0.68524676	null
		GO:004529	mRNA cis splicing, via spliceosome	6	2	0.2	0.015	-1.823908741	4.191367166	0.051675544	0.885690706	0.45461677	null
		GO:004593	positive regulation of nucleobase-containing compound metabolic process	72	4	2.39	0.03302	-1.481222931	5.411979785	0.858863101	0.892541765	0.27565194	null

		GO:000989 positive regulation of catabolic process	14	2	0.46	0.03303	-1.481091426	4.828324703	0.224008293	0.899854518	0.77312404	GO:0045935
		GO:004851 negative regulation of biological process	183	9	6.06	0.03277	-1.484523559	5.867307176	2.450487139	0.926700309	0.25932672	null
		GO:005079 regulation of catalytic activity	117	11	3.88	0.02772	-1.557206774	5.306945868	0.67435653	0.902356791	0	null
		GO:001095 negative regulation of endopeptidase activity	9	2	0.3	0.03373	-1.471983659	3.82314806	0.022132406	0.883949092	0.71091461	GO:0050790
		GO:005124 negative regulation of multicellular organismal process	16	2	0.53	0.03305	-1.480828536	4.677743789	0.158372769	0.919914969	0.1972746	null
		GO:005170 biological process involved in interaction with symbiont	10	2	0.33	0.04126	-1.384470776	3.692229836	0.016371461	0.963215993	0.59405826	null
		GO:007098 demethylation	7	2	0.23	0.03299	-1.481617684	4.410827828	0.085655806	0.983571209	0.02912877	null
		GO:009040 organophosphate biosynthetic process	108	6	3.58	0.00048	-3.318758763	6.151678068	4.716540884	0.849641573	0	null
**T2**	**Heat,**	**"magenta" module**										
	**Combined**	GO:000218 cytoplasmic translation	12	2	0.14	0.0077	-2.113509275	4.558924464	0.120464287	0.906474611	0.00699101	null
		GO:000659 polyamine metabolic process	7	2	0.08	0.0111	-1.954677021	4.571254506	0.123933493	0.932413493	0.12568239	null
		GO:000905 biosynthetic process	1798	24	20.29	0.0312	-1.505845406	6.928894185	28.23839283	0.972950907	0.03690011	null
		GO:000979 embryo development ending in seed dormancy	55	3	0.62	0.0237	-1.625251654	3.785116195	0.020276398	1	0	null
		GO:001656 protein ubiquitination	224	6	2.53	0.0395	-1.403402904	5.395139353	0.82619668	0.90623159	0.23697339	null
		GO:004225 ribosome biogenesis	59	3	0.67	0.0225	-1.647817482	5.696511029	1.653697223	0.824696707	0.00903128	null
		GO:002261 protein-RNA complex assembly	30	3	0.34	0.0244	-1.612610174	5.020663692	0.348823224	0.825398573	0.79948034	GO:0042254
		GO:005130 regulation of cell division	10	2	0.11	0.0053	-2.27572413	4.428992812	0.089314605	1	0	null
		GO:007058 calcium ion transmembrane transport	8	2	0.09	0.0034	-2.468521083	4.897654577	0.262781581	0.937062095	0.26194396	null
		GO:199054 mitochondrial transmembrane transport	8	2	0.09	0.0034	-2.468521083	4.32467335	0.070242284	0.937647093	0	null
**T2**	**Combined**	**"pink" module**										
		GO:000628 nucleotide-excision repair	5	1	0.04	0.0397	-1.401209493	4.878705585	0.251562374	0.829310687	0.25233873	null
		GO:000633 chromatin remodeling	35	2	0.28	0.0319	-1.496209317	5.400743432	0.836926939	0.928233931	0.67612431	null
		GO:000639 mRNA processing	117	5	0.94	0.01	-2	5.491773378	1.032090592	0.845598638	0.42838212	null
		GO:000641 translational initiation	53	3	0.43	0.0087	-2.060480747	5.194211757	0.520181402	0.853392952	0.22610919	null
		GO:000838 RNA splicing	104	4	0.84	0.0185	-1.732828272	5.335285352	0.719828747	0.848591236	0.64689361	null
		GO:000991 positive regulation of flower development	10	2	0.08	0.0027	-2.568636236	2.587710965	0.001283906	0.759443679	0	null
		GO:004858 positive regulation of post-embryonic development	16	3	0.13	0.0454	-1.342944147	3.265525335	0.006126825	0.777437695	0.75540737	GO:0009911
		GO:200024 positive regulation of reproductive process	13	3	0.1	0.023	-1.638272164	3.849665055	0.023526076	0.802110748	0.72385277	GO:0009911
		GO:001007 regulation of meristem growth	5	1	0.04	0.0397	-1.401209493	2.993876915	0.003276288	0.801913642	0.40715223	null
		GO:001022 vegetative to reproductive phase transition of meristem	30	4	0.24	0.0025	-2.602059991	3.30920418	0.00677543	0.902446018	0	null
		GO:001657 histone modification	43	3	0.35	0.0216	-1.665546249	5.052155067	0.375056811	0.905362474	0.36564459	null
		GO:001802 peptidyl-lysine methylation	13	2	0.1	0.0079	-2.102372909	4.519512983	0.110013427	0.911216876	0	null
		GO:002261 protein-RNA complex assembly	30	2	0.24	0.024	-1.619788758	5.020663692	0.348823224	0.928639381	0	null
		GO:003288 regulation of polysaccharide biosynthetic process	6	1	0.05	0.0474	-1.324221658	3.40277707	0.008405259	0.905954273	0.22099952	null
		GO:003424 regulation of transcription elongation by RNA polymerase II	5	1	0.04	0.0397	-1.401209493	3.973543469	0.031292708	0.898165216	0.12071471	null
		GO:004474 protein transmembrane import into intracellular organelle	5	1	0.04	0.0397	-1.401209493	4.486657131	0.101997332	0.994523511	0.00779128	null
		GO:004594 positive regulation of transcription by RNA polymerase II	24	2	0.19	0.0157	-1.804100348	5.045412958	0.369279235	0.79215114	0.45063054	null
		GO:004857 long-day photoperiodism, flowering	12	2	0.1	0.0389	-1.410050399	2.378397901	0.000791631	0.814976253	0.61833485	null
		GO:005160 defense response to virus	6	1	0.05	0.0474	-1.324221658	4.673048568	0.156669764	0.916290962	0.46072997	null
		GO:007148 cellular response to blue light	5	1	0.04	0.0397	-1.401209493	3.660486016	0.015217277	0.890615325	0.00676539	null
		GO:009750 mannosylation	6	1	0.05	0.0474	-1.324221658	4.024485668	0.035187666	0.986886201	0.01995905	null
**T2**	**Shade**	**"royalblue" module**										
		GO:000603 chitin catabolic process	8	1	0.02	0.02271	-1.643782866	4.254040258	0.059698291	0.643403473	0.50779323	null
		GO:000695 defense response	322	5	0.92	0.00042	-3.37675071	5.495515199	1.041021388	1	0	null
		GO:001015 leaf senescence	10	1	0.03	0.02831	-1.548060131	3.66996737	0.015553221	1	0	null
		GO:001699 cell wall macromolecule catabolic process	11	1	0.03	0.0311	-1.507239611	4.614137591	0.136795834	0.659723144	0.53863378	null
		GO:003140 oxylipin biosynthetic process	13	1	0.04	0.03666	-1.435807539	3.632659713	0.014272641	0.925310217	0.14630527	null
		GO:004240 biogenic amine biosynthetic process	10	1	0.03	0.02831	-1.548060131	4.989605531	0.324748328	0.888712208	0.03397279	null
		GO:004549 pectin catabolic process	30	2	0.09	0.0032	-2.494850022	4.412729273	0.086031664	0.664849558	0	null
**T2**	**Combined**	**"turquoise" module**										
		GO:000000 reproduction	280	22	12.64	0.04355	-1.361011841	5.312792806	0.683496875	1	0	null
		GO:000608 acetyl-CoA biosynthetic process from pyruvate	8	2	0.36	0.04746	-1.323672266	4.335457901	0.072008486	0.688615633	0.54482304	null
		GO:000613 nucleobase-containing compound metabolic process	1792	88	80.89	0.02538	-1.595508382	6.851645188	23.63692449	0.788184999	0.32282979	null
		GO:000636 transcription by RNA polymerase II	74	7	3.34	0.03613	-1.442132038	4.962781175	0.305296823	0.772456443	0.33188054	null
		GO:000640 mRNA catabolic process	27	5	1.22	0.01129	-1.947306058	4.945936111	0.2936818	0.712660376	0.68905201	null
		GO:000989 negative regulation of metabolic process	125	8	5.64	0.04488	-1.347947152	5.704449957	1.684204952	0.938059887	0.729854	GO:0006402

		GO:001714	negative regulation of translation	10	2	0.45	0.04503	-1.346498053	4.796074538	0.207976102	0.936641674	0.83244555	GO:0006402
		GO:003465	nucleobase-containing compound catabolic process	38	7	1.72	0.04621	-1.335264031	5.397886153	0.831438741	0.783975087	0.85200628	GO:0006402
		GO:000641	tRNA aminoacylation for protein translation	12	3	0.54	0.01476	-1.830913643	5.466756439	0.97431816	0.669575626	0.28811995	null
		GO:000803	tRNA processing	38	5	1.72	0.0444	-1.35261703	5.638987162	1.448545049	0.787252325	0.80876927	GO:0006418
		GO:000645	regulation of translational fidelity	6	2	0.27	0.02699	-1.568797115	4.917027365	0.274769136	0.974431227	0	null
		GO:000648	protein N-linked glycosylation	8	2	0.36	0.04746	-1.323672266	4.505584056	0.106540894	0.761287863	0.66936153	null
		GO:000650	GPI anchor biosynthetic process	10	3	0.45	0.00861	-2.064996849	4.696819273	0.165484143	0.716906637	0.3780953	null
		GO:000865	phospholipid biosynthetic process	42	5	1.9	0.03628	-1.440332722	5.552614686	1.187296837	0.750028956	0.77526225	GO:0006506
		GO:000651	ubiquitin-dependent protein catabolic process	107	13	4.83	0.00531	-2.274905479	5.375253812	0.789219529	0.801748155	0.26174252	null
		GO:000654	glutamine metabolic process	8	2	0.36	0.04746	-1.323672266	5.263851387	0.610653517	0.79063767	0.56485443	null
		GO:000673	one-carbon metabolic process	16	4	0.72	0.0469	-1.328827157	5.018409207	0.347017108	0.840039732	0.31101493	null
		GO:000689	post-Golgi vesicle-mediated transport	8	2	0.36	0.04746	-1.323672266	4.236864622	0.05738327	0.910412398	0.24997922	null
		GO:000905	macromolecule biosynthetic process	1318	50	59.49	0.04759	-1.322484295	6.661562823	15.25838504	0.791245524	0.56953202	null
		GO:000911	vitamin biosynthetic process	18	2	0.81	0.0451	-1.345823458	5.559318793	1.205767118	0.775650451	0.35382163	null
		GO:000920	purine ribonucleoside triphosphate biosynthetic process	14	4	0.63	0.01133	-1.94577009	5.145134226	0.464597596	0.673769163	0.49424174	null
		GO:000616	purine nucleotide metabolic process	60	13	2.71	0.04345	-1.362010219	5.869704491	2.464051304	0.651120296	0.90676201	GO:0009206
		GO:000618	IMP biosynthetic process	5	2	0.23	0.01854	-1.73189027	5.113585747	0.432044265	0.655149933	0.81692263	GO:0009206
		GO:000914	purine nucleoside triphosphate metabolic process	15	5	0.68	0.04453	-1.351347305	5.200215549	0.527422497	0.707620344	0.91151638	GO:0009206
		GO:000915	purine ribonucleotide biosynthetic process	35	9	1.58	0.01399	-1.854182286	5.679419719	1.58988112	0.613899189	0.722052	GO:0009206
		GO:000955	pollen development	41	6	1.85	0.00749	-2.125518182	3.65829765	0.015140775	0.869247699	0.56451806	null
		GO:000979	post-embryonic development	240	20	10.83	0.04055	-1.392009141	4.666826744	0.154441223	0.851224125	0.66473909	null
		GO:000988	embryonic pattern specification	5	2	0.23	0.01854	-1.73189027	3.689308859	0.016261698	0.868748493	0.56714766	null
		GO:000988	tissue development	99	11	4.47	0.02601	-1.584859648	5.098172841	0.416979993	0.84979273	0.63005439	null
		GO:001001	meristem initiation	5	2	0.23	0.01854	-1.73189027	2.707570176	0.001693026	0.889002763	0.43287153	null
		GO:001503	protein transport	180	23	8.12	0.00103	-2.987162775	5.926497304	2.808294377	0.827552859	0	null
		GO:000661	protein targeting to membrane	11	3	0.5	0.02683	-1.571379327	4.71092087	0.170945732	0.832585266	0.91736592	GO:0015031
		GO:006500	intracellular protein transmembrane transport	13	4	0.59	0.00426	-2.370590401	4.884455496	0.254915164	0.843774922	0.72083406	GO:0015031
		GO:009015	establishment of protein localization to membrane	17	5	0.77	0.02648	-1.577082019	4.990849228	0.325679658	0.842266689	0.7398233	GO:0015031
		GO:001619	endosomal transport	16	4	0.72	0.01833	-1.736837535	4.686841751	0.161725559	0.839205925	0.63246062	null
		GO:001657	histone modification	43	9	1.94	0.00023	-3.638272164	5.052155067	0.375056811	0.843797407	0	null
		GO:002170	developmental maturation	37	5	1.67	0.01133	-1.94577009	4.250371202	0.059196038	0.868696887	0.52448876	null
		GO:003142	chloroplast RNA processing	16	3	0.72	0.03291	-1.482672118	2.957128198	0.003010194	0.872185122	0.07387727	null
		GO:003150	protein-containing complex localization	7	2	0.32	0.04501	-1.346690987	4.395326393	0.082652265	0.936122913	0.25444606	null
		GO:003472	piecemeal microautophagy of the nucleus	5	2	0.23	0.01854	-1.73189027	3.254548077	0.005973821	0.892186016	0.63827569	null
		GO:003621	protein modification process	820	42	37.01	0.0461	-1.336299075	6.403188836	8.416557701	0.790894451	0.5822516	null
		GO:004239	cellular modified amino acid biosynthetic process	8	2	0.36	0.04746	-1.323672266	5.130176107	0.448868087	0.794600501	0.32685317	null
		GO:003599	tetrahydrofolate interconversion	8	2	0.36	0.04746	-1.323672266	4.661254083	0.152472124	0.783958899	0.83232956	GO:0042398
		GO:004341	macromolecule methylation	51	7	2.3	0.02696	-1.569280112	5.594848707	1.308559407	0.877017518	0.39969794	null
		GO:004343	oxoacid metabolic process	254	25	11.46	0.02015	-1.69572495	6.420406157	8.7569291	0.758689035	0.62391026	null
		GO:004424	cellular biosynthetic process	1712	82	77.27	0.02198	-1.657972312	6.865251598	24.38919015	0.787289704	0.66782618	null
		GO:004504	protein targeting to ER	7	3	0.32	0.00202	-2.694648631	4.672744198	0.15656	0.844503124	0.68580638	null
		GO:004516	cell fate commitment	20	4	0.9	0.04696	-1.328271912	4.304598226	0.067069108	0.859742496	0.52919514	null
		GO:004611	nucleobase biosynthetic process	7	2	0.32	0.03667	-1.43568909	4.978148549	0.316293176	0.7066328	0.53137748	null
		GO:004628	anthocyanin-containing compound metabolic process	17	2	0.77	0.04509	-1.345919765	3.015778756	0.003445923	0.968479608	0.02738989	null
		GO:004819	Golgi vesicle transport	42	6	1.9	0.04661	-1.331520897	5.095950031	0.414851237	0.898825878	0.68050521	null
		GO:004858	regulation of long-day photoperiodism, flowering	7	2	0.32	0.03667	-1.43568909	3.055378331	0.003775215	0.979678288	0.14042442	null
		GO:004858	developmental growth	59	7	2.66	0.03595	-1.444301105	4.413383597	0.086161385	0.865834393	0.5388959	null
		GO:004860	reproductive structure development	204	18	9.21	0.00258	-2.588380294	4.608044406	0.134889933	0.852377493	0	null
		GO:005065	RNA transport	18	2	0.81	0.0451	-1.345823458	4.78879743	0.2045202	0.880618098	0.64993427	null
		GO:006191	selective autophagy	5	2	0.23	0.01854	-1.73189027	3.778006461	0.019947106	0.884621763	0.39421884	null
		GO:007089	transcription preinitiation complex assembly	8	2	0.36	0.04746	-1.323672266	4.192149125	0.051768677	0.802404028	0.3678924	null
		GO:009750	mannosylation	6	2	0.27	0.02699	-1.568797115	4.024485668	0.035187666	0.951278964	0.02176723	null
		GO:190157	organic substance biosynthetic process	1746	82	78.81	0.00035	-3.455931956	6.910651347	27.07678741	0.803799069	0.30564683	null
**T2**	**Combined**	**"yellow" module**											
		GO:000007	regulation of cyclin-dependent protein serine/threonine kinase activity	9	2	0.33	0.0411	-1.386158178	4.324323619	0.070185739	0.917606192	0.28885877	null
		GO:000645	protein folding	67	6	2.47	0.0362	-1.441291429	5.502977151	1.059062592	0.989850438	0.01028863	null
		GO:000654	glutamine metabolic process	8	2	0.3	0.0328	-1.484126156	5.263851387	0.610653517	0.867392191	0.5356103	null
		GO:000655	L-phenylalanine metabolic process	7	2	0.26	0.0252	-1.598599459	4.475133434	0.099326408	0.876312733	0.13305992	null
		GO:000687	intracellular calcium ion homeostasis	8	2	0.3	0.0328	-1.484126156	4.336819829	0.072234667	1	0	null
		GO:000690	vesicle fusion	5	2	0.18	0.0126	-1.899629455	3.966188681	0.030767172	0.922382648	0.00749651	null
		GO:000715	cell adhesion	8	3	0.3	0.0024	-2.619788758	5.326909079	0.706078316	0.990199412	0	null
		GO:000906	amino acid catabolic process	17	3	0.63	0.023	-1.638272164	5.328267447	0.708290226	0.817068559	0.679041	null

		GO:000924	glycolipid biosynthetic process	11	2	0.41	0.0368	-1.434152181	5.050963322	0.374029021	0.876615348	0.2204724	null
		GO:000926	response to temperature stimulus	81	7	2.99	0.0015	-2.823908741	4.805602793	0.212589514	0.929070656	0	null
		GO:000961	response to virus	14	3	0.52	0.0325	-1.488116639	4.714983225	0.172552277	0.89564432	0.50126013	null
		GO:000974	gibberellic acid mediated signaling pathway	16	4	0.59	0.0323	-1.490797478	3.461048092	0.009612662	0.866237524	0.21261324	null
		GO:000993	negative regulation of gibberellic acid mediated signaling pathway	6	2	0.22	0.0184	-1.735182177	2.334453751	0.000715129	0.908634136	0.46954313	null
		GO:001022	inflorescence development	8	2	0.3	0.0328	-1.484126156	2.530199698	0.001124249	1	0	null
		GO:001027	hydrotropism	7	2	0.26	0.0252	-1.598599459	3.418301291	0.008711267	0.908233373	0.21126278	null
		GO:001603	cellular response to phosphate starvation	7	2	0.26	0.0252	-1.598599459	3.542825427	0.011605045	0.884166028	0.42540078	null
		GO:001605	carbohydrate biosynthetic process	99	8	3.65	0.0401	-1.396855627	5.460651322	0.960717407	0.896358239	0.61009148	null
		GO:001605	carbohydrate catabolic process	88	8	3.25	0.0039	-2.408935393	5.599383018	1.322293207	0.873202144	0	null
		GO:001655	mRNA modification	19	4	0.7	0.0248	-1.605548319	4.085183508	0.040466315	0.947212554	0.06623472	null
		GO:002260	regulation of cell morphogenesis	9	2	0.33	0.0411	-1.386158178	5.332721164	0.715591193	0.920989169	0.22139099	null
		GO:003164	regulation of protein stability	5	2	0.18	0.0126	-1.899629455	4.194680813	0.052071359	0.934424854	0.17157609	null
		GO:003250	DNA duplex unwinding	9	2	0.33	0.0411	-1.386158178	5.228295109	0.562650077	0.938649091	0.48567709	null
		GO:003254	mitochondrial translation	9	2	0.33	0.0411	-1.386158178	4.348265911	0.074163851	0.912227883	0.18570525	null
		GO:003497	response to endoplasmic reticulum stress	20	3	0.74	0.0183	-1.73754891	4.571825249	0.124096476	0.908822534	0.25495204	null
		GO:004581	negative regulation of gene expression, epigenetic	9	2	0.33	0.0368	-1.434152181	4.235730397	0.057233592	0.859409034	0.30278339	null
		GO:004594	positive regulation of transcription by RNA polymerase II	24	4	0.89	0.0107	-1.970616222	5.045412958	0.369279235	0.908850211	0	null
		GO:004603	ADP metabolic process	19	2	0.7	0.0368	-1.434152181	3.503245771	0.010593886	0.85559386	0.43490843	null
		GO:004617	polyol catabolic process	5	2	0.18	0.0126	-1.899629455	4.4130985	0.08610484	0.875587203	0.47564895	null
		GO:007058	calcium ion transmembrane transport	8	2	0.3	0.0328	-1.484126156	4.897654577	0.262781581	0.96295587	0.27136656	null
		GO:008013	regulation of cellular response to stress	5	2	0.18	0.0126	-1.899629455	4.685992393	0.161409572	0.904836413	0.21030641	null
		GO:009040	organophosphate biosynthetic process	108	11	3.98	0.008	-2.096910013	6.151678068	4.716540884	0.860378264	0.05886897	null
		GO:000865	phospholipid biosynthetic process	42	4	1.55	0.025	-1.602059991	5.552614686	1.187296837	0.841957058	0.70901566	GO:0090407
		GO:014011	export across plasma membrane	6	2	0.22	0.0184	-1.735182177	4.523759469	0.111094436	0.964198822	0.18528497	null
**T3**	**Shade**	**"greenyellow" module**											
		GO:000990	chloroplast accumulation movement	8	1	0.03	0.028	-1.552841969	1.672097858	0.000153004	0.675584093	0.31731593	null
		GO:000990	chloroplast avoidance movement	9	1	0.03	0.032	-1.494850022	1.86332286	0.000239485	0.672450313	0.94610681	GO:0009904
		GO:003310	mitochondrial respiratory chain complex assembly	8	1	0.03	0.028	-1.552841969	4.474667812	0.099219971	0.81120988	0.0076442	null
		GO:003420	lipid translocation	9	1	0.03	0.032	-1.494850022	4.393452355	0.082296363	0.594489858	0.33591842	null
		GO:001591	phospholipid transport	10	1	0.04	0.035	-1.455931956	4.560098007	0.120790253	0.761803487	0.76854024	GO:0034204
		GO:003530	regulation of protein dephosphorylation	8	1	0.03	0.028	-1.552841969	4.21285319	0.054296574	0.881902634	0.18139956	null
		GO:004277	ATP synthesis coupled electron transport	6	1	0.02	0.021	-1.677780705	4.92781162	0.281677614	0.993189896	0	null
		GO:005105	regulation of DNA metabolic process	12	1	0.04	0.042	-1.37675071	4.836330446	0.228175997	0.874848877	0.27318285	null
		GO:005160	defense response to virus	6	1	0.02	0.021	-1.677780705	4.673048568	0.156669764	1	0	null
		GO:007180	potassium ion transmembrane transport	14	1	0.05	0.049	-1.30980392	5.1261444	0.44472034	0.853840043	0.0086601	null
		GO:008013	regulation of cellular response to stress	5	1	0.02	0.018	-1.744727495	4.685992393	0.161409572	0.892930578	0	null
**T3**	**Combined**	**"salmon" module**											
		GO:000630	DNA methylation	7	1	0.04	0.0358	-1.446116973	4.734415753	0.18044863	0.76060182	0.65187592	null
		GO:000641	translational initiation	53	2	0.28	0.0305	-1.515700161	5.194211757	0.520181402	0.807113587	0.22610919	null
		GO:000687	intracellular calcium ion homeostasis	8	1	0.04	0.0408	-1.389339837	4.336819829	0.072234667	1	0	null
		GO:001022	vegetative to reproductive phase transition of meristem	30	2	0.16	0.0104	-1.982966661	3.30920418	0.00677543	1	0	null

		GO:00180‹	peptidyl-lysine methylation	13	2	0.07	0.005	-2.301029996	4.519512983	0.110013427	0.802664098	0	null
		GO:00311‹	mRNA 3'-end processing	9	1	0.05	0.0458	-1.339134522	4.368955945	0.077782736	0.807534226	0.33919138	null
		GO:00342‹	regulation of transcription elongation by RNA polymerase II	5	1	0.03	0.0257	-1.590066877	3.973543469	0.031292708	0.945546264	0.30368765	null
		GO:00436‹	RNA polyadenylation	9	1	0.05	0.0458	-1.339134522	4.037745129	0.036278653	0.847287269	0.08020877	null
		GO:00459‹	positive regulation of transcription by RNA polymerase II	24	2	0.12	0.0067	-2.173925197	5.045412958	0.369279235	0.945546264	0	null
		GO:00513‹	cell division	68	2	0.35	0.0481	-1.317854924	5.61902129	1.383458345	0.991965606	0	null
		GO:00708‹	transcription preinitiation complex assembly	8	1	0.04	0.0408	-1.389339837	4.192149125	0.051768677	0.824913965	0.24845084	null
		GO:00975‹	mannosylation	6	1	0.03	0.0308	-1.511449283	4.024485668	0.035187666	0.97830599	0.01995905	null
**T3**	**Combined**	**"darkgrey" module**											
		GO:00065‹	glutamine metabolic process	8	1	0.01	0.0114	-1.943095149	5.263851387	0.610653517	0.96740662	0	null
		GO:00070‹	vacuole organization	16	1	0.02	0.0227	-1.643974143	4.38313288	0.080363852	0.996520746	0.00799624	null
		GO:00098‹	pollen germination	9	1	0.01	0.0128	-1.89279003	3.020775488	0.003485837	0.805218064	0	null
		GO:00488‹	pollen tube development	16	1	0.02	0.0227	-1.643974143	3.367355921	0.007746675	0.709415474	0.92394507	GO:0009846
		GO:00105‹	regulation of starch biosynthetic process	5	1	0.01	0.0071	-2.148741651	2.245512668	0.000582082	0.970965475	0	null
		GO:00311‹	SCF-dependent proteasomal ubiquitin-dependent protein catabolic process	19	1	0.03	0.0269	-1.57024772	4.092650478	0.041168139	0.97115291	0.13076183	null
		GO:00342‹	carbohydrate transmembrane transport	15	1	0.02	0.0213	-1.671620397	5.040950269	0.36550402	0.946019132	0.00912714	null
		GO:00430‹	regulation of programmed cell death	8	1	0.01	0.0114	-1.943095149	5.012567288	0.342380412	0.970965475	0.13161777	null
		GO:00486‹	anatomical structure formation involved in morphogenesis	35	1	0.05	0.0491	-1.308918508	4.915331229	0.273698106	0.892890091	0	null
		GO:00516‹	organelle localization	21	1	0.03	0.0297	-1.527243551	4.776737495	0.198918911	0.949654045	0.22948248	null
**T3**	**Heat,**	**"darkred" module**											
	**Shade,**	GO:00066‹	protein targeting to mitochondrion	7	1	0.02	0.022	-1.657577319	4.44345075	0.092338103	0.890610819	0.59213251	null
	**Combined**	GO:00068‹	nitrogen compound metabolic process	3189	10	10.28	0.037	-1.431798276	7.166808942	48.83764952	0.993066675	0.03819961	null
		GO:00097‹	brassinosteroid mediated signaling pathway	13	1	0.04	0.041	-1.387216143	3.724357804	0.017628758	0.616539245	0.50865968	null
		GO:00101‹	lateral root morphogenesis	8	1	0.03	0.026	-1.585026652	2.80685803	0.002128756	1	0	null
		GO:00101‹	response to nitrate	6	1	0.02	0.019	-1.721246399	3	0.003322855	0.608521754	0.63823194	null
		GO:19016‹	cellular response to nitrogen compound	7	1	0.02	0.022	-1.657577319	4.644123341	0.146574806	0.606540662	0.70000138	GO:0010167
		GO:00102‹	response to chitin	7	1	0.02	0.022	-1.657577319	2.481442629	0.001004507	0.665016639	0.32728913	null
		GO:00421‹	regulation of cell population proliferation	5	1	0.02	0.016	-1.795880017	4.856644319	0.239102501	0.941275221	0.15551851	null
		GO:00450‹	protein import into chloroplast stroma	5	1	0.02	0.016	-1.795880017	2.761927838	0.001919206	0.891586175	0.00513723	null
		GO:00465‹	S-adenosylmethionine metabolic process	6	1	0.02	0.019	-1.721246399	4.439000924	0.091396794	0.98720716	0.00644595	null
		GO:00712‹	cellular response to inorganic substance	5	1	0.02	0.016	-1.795880017	3.931152639	0.0283823	0.670595134	0	null
		GO:00906‹	activation of GTPase activity	5	1	0.02	0.016	-1.795880017	3.793301354	0.020662235	0.948434681	0	null
**T3**	**Heat,**	**"magenta" module**											
	**Shade,**	GO:00003‹	RNA splicing, via transesterification reactions	77	2	0.55	0.0071	-2.148741651	5.192369495	0.51797947	0.828011738	0	null
	**Combined**	GO:00452‹	mRNA cis splicing, via spliceosome	6	1	0.04	0.0422	-1.374687549	4.191367166	0.051675544	0.83311641	0.7635294	GO:0000375
		GO:00064‹	regulation of translation	31	2	0.22	0.0204	-1.690369833	5.640985006	1.45522402	0.852405814	0	null
		GO:00064‹	regulation of translational fidelity	6	1	0.04	0.0422	-1.374687549	4.917027365	0.274769136	0.890453933	0.22299092	null
		GO:00094‹	response to water deprivation	44	2	0.32	0.0392	-1.406713933	3.798857732	0.020928329	0.787325659	0.29713445	null
		GO:00096‹	salicylic acid metabolic process	5	1	0.04	0.0353	-1.452225295	3.06669855	0.003875001	0.945410662	0.12706844	null
		GO:00102‹	response to chitin	7	1	0.05	0.0491	-1.308918508	2.481442629	0.001004507	0.77548178	0.65356468	null
		GO:00106‹	positive regulation of gene expression	6	1	0.04	0.0422	-1.374687549	4.984846729	0.321209272	0.863000299	0.37448759	null
		GO:00464‹	phosphatidylcholine metabolic process	5	1	0.04	0.0353	-1.452225295	4.128786859	0.044740457	0.9671584	0.05651846	null
		GO:00512‹	regulation of multicellular organismal process	65	2	0.47	0.0484	-1.315154638	5.206480461	0.535086018	0.883201265	0.24668064	null
		GO:00704‹	trehalose metabolism in response to stress	5	1	0.04	0.0353	-1.452225295	2.77815125	0.001992382	0.863931052	0.03512493	null
		GO:19016‹	cellular response to nitrogen compound	7	1	0.05	0.0491	-1.308918508	4.644123341	0.146574806	0.773469735	0.42938691	null
**T3**	**Heat**	**"Sienna 3" module**											
		GO:00021‹	cytoplasmic translation	12	1	0.01	0.0107	-1.970616222	4.558924464	0.120464287	0.837400821	0	null
		GO:00064‹	translational initiation	53	1	0.05	0.0466	-1.331614083	5.194211757	0.520181402	0.827790539	0.47198297	null
		GO:00068‹	iron ion transport	12	1	0.01	0.0107	-1.970616222	4.936820254	0.287581585	0.856236454	0.25255471	null
		GO:00068‹	intracellular iron ion homeostasis	5	1	0	0.0045	-2.346787486	4.526067758	0.111686496	1	0	null
		GO:00100‹	response to metal ion	22	1	0.02	0.0196	-1.707743929	4.504593182	0.106298083	1	0	null
		GO:00226‹	protein-RNA complex assembly	30	1	0.03	0.0266	-1.575118363	5.020663692	0.348823224	0.993065621	0.00789601	null
		GO:00356‹	oligopeptide transmembrane transport	18	1	0.02	0.016	-1.795880017	4.328522306	0.070867606	0.864063098	0.22385023	null
		GO:00458‹	negative regulation of DNA-templated transcription	35	1	0.03	0.031	-1.508638306	5.339554785	0.726940121	1	0	null
		GO:00516‹	protein maturation	23	1	0.02	0.0204	-1.690369833	5.213639927	0.543980225	0.854685521	0.3528939	null
		GO:00516‹	establishment of localization in cell	162	2	0.15	0.0088	-2.055517328	5.818033785	2.187655665	0.833539586	0.30846836	null
		GO:00986‹	import into cell	6	1	0.01	0.0054	-2.26760624	5.275398896	0.627108133	0.849185599	0	null
